# Rotator Cuff-Related Shoulder Pain: A Survey of Current Physiotherapy Practice in Cyprus

**DOI:** 10.3390/clinpract16010011

**Published:** 2026-01-04

**Authors:** George M. Pamboris, Spyridon Plakias, Charalambos Papacharalambous, Kyriakos Pavlou, Andrew Smythe, Anna Christakou, Eleftherios Paraskevopoulos

**Affiliations:** 1 Department of Health Sciences, School of Sciences, European University Cyprus, Nicosia 2404, Cyprus; c.papacharalambous@euc.ac.cy (C.P.); k.pavlou@external.euc.ac.cy (K.P.); 2 Department of Physical Education and Sport Science, University of Thessaly, 42150 Trikala, Greece; spyros_plakias@yahoo.gr; 3 Physiotherapy Department, School of Primary and Allied Health Care, Faculty of Medicine, Nursing and Health Sciences, Monash University, Frankston, VIC 3199, Australia; andy.smythe@monash.edu; 4 Biomechanics Laboratory, Department of Physiotherapy, University of Peloponnese, 23100 Sparta, Greece; a.christakou@go.uop.gr; 5 School of Physical Education and Sports Science, National and Kapodistrian University of Athens, 17237 Dafne, Greece; elparaskev@phed.uoa.gr

**Keywords:** Cyprus, rotator cuff, shoulder pain, physiotherapy management, evidence-based practice

## Abstract

**Background:** Rotator cuff-related shoulder pain (RCRSP) is a prevalent musculoskeletal disorder treated by physiotherapists. Although international guidelines support active, exercise-based management, little is known about current physiotherapy practices in Cyprus. **Aim:** To investigate physiotherapy management practices for RCRSP in Cyprus, assess adherence to recommended clinical guidelines, and compare findings with practices in other countries. **Methods:** A cross-sectional online survey was conducted among Cypriot physiotherapists between June and July 2024. An English case vignette-based questionnaire, adapted from validated international surveys, examined demographics, clinical reasoning, treatment preferences, and guideline adherence. Descriptive statistics summarised responses; chi-square tests and logistic regression identified associations between demographics and clinical decisions. Content analysis was performed on open-ended responses. **Results:** A total of 143 physiotherapists completed the survey. Most adhered to guideline-recommended care, with 99.3% (n = 142/143) prescribing exercise and 100% (n = 143/143) providing patient education. Conservative management was preferred, with 64.3% (n = 91/143) not recommending imaging, 72.0% (n = 103/143) not recommending injections, and 73.4% (n = 104/143) not recommending surgical referrals. Significant associations were found between special interest in shoulder conditions and recommendations for surgery (χ^2^ = 4.937, *p* = 0.026) and injections (χ^2^ = 9.143, *p* = 0.002). Physiotherapists recommending surgery were nearly seven times more likely to suggest MRI (Exp(B) = 6.944, *p* < 0.001). **Conclusions:** Cypriot physiotherapists predominantly use exercise and education for the management of RCRSP, aligning closely with international recommendations. Conservative strategies were favoured, with limited use of imaging, injections, and surgical referrals. However, variation in clinical decision-making, particularly regarding referrals and imaging, indicates partial adherence to best practice and highlights opportunities for enhanced guideline implementation and targeted clinical training.

## 1. Introduction

Shoulder pain is a common musculoskeletal condition [1,2] that may cause functional limitations [3], inability to work, and a decrease in quality of life [4,5] and affect sports performance [6,7], causing premature sports career termination [8], and is associated with a substantial economic burden [5]. This condition is the third most common musculoskeletal condition presented to healthcare professionals in primary care settings, followed or preceded by lumbar and cervical pain [4,9]. Additionally, it affects a large segment of the population, with studies showing that 40% to 54% of individuals continue to have symptoms for one to three years. [10]. A study conducted in New Zealand reported that shoulder pain was the most common musculoskeletal problem among participants over the age of 65 [11].

There are myriad causes of shoulder pain; however, recent literature advocates shifting away from conventional diagnostic labels based on tissue-specific identification, as special tests commonly used in clinical practice have demonstrated low validity and diagnostic accuracy [12]. This transition is essential because clinical symptoms frequently do not align with imaging results from techniques like magnetic resonance imaging (MRI) and ultrasound, which can result in misdiagnosis and ineffective treatment strategies [13,14]. On the other hand, rotator cuff-related shoulder pain (RCRSP) is an umbrella term that includes various shoulder conditions, such as subacromial pain syndrome, impingement syndrome, rotator cuff tendinopathy, and subacromial bursitis, which share overlapping symptoms but have distinct mechanisms [15,16]. Due to the challenges in precisely identifying affected tissue, clinicians and researchers are increasingly adopting RCRSP as a diagnostic term instead of traditional labels like impingement syndrome, which can misleadingly imply a singular mechanical cause [17]. This shift aligns with contemporary research advocating for a multifactorial approach to diagnosis and management [18,19,20].

International guidelines recommend conservative management as the primary treatment approach, which includes lifestyle and activity modifications, patient education, pain relief, and physiotherapy-led exercise rehabilitation. These conservative management practices include exercise programs for the rotator cuff and the scapular region, manual therapy techniques [21,22,23], while a wide variety of other physiotherapy methods, such as physical modalities, are not supported by the literature [22,24,25,26]. Exercise therapy and education are recommended in evidence-based practice as the principal non-surgical approach for 6–12 weeks for initial management prior to considering imaging, injection or surgical referral [16,18,19,20,25,27,28]. Exercise therapy results in outcomes comparable to more invasive treatment options, such as subacromial decompression surgery [29,30], as well as corticosteroid [31] and platelet-rich plasma injections [32].

Research on physiotherapy practices for managing RCRSP in the United Kingdom [33,34], Belgium and the Netherlands [35], Australia [36], France [37], Italy [38], and Greece [39] has used heterogeneous survey designs, with sample sizes ranging from approximately 120 to 550 physiotherapists and response rates frequently below 20%. These studies relied predominantly on convenience sampling and self-reported behaviour, limiting generalisability and introducing potential selection and reporting biases. Most did not include cross-national methodological harmonisation, making direct comparison challenging. The present study distinguishes itself by applying a standardised questionnaire structure adapted from validated international surveys, enabling methodologically consistent comparison while addressing an unexplored geographical context, Cyprus, where no prior data exist on RCRSP management or guideline adherence.

Although this is the first study to investigate physiotherapy management of RCRSP in Cyprus, several contextual factors highlight the need for such research. Cyprus operates within a mixed public–private healthcare system under the General Healthcare System (GESY) [40], where physiotherapists differ substantially in training background, postgraduate education, and access to continuing professional development. Despite these structural characteristics, no empirical data currently exist describing how Cypriot physiotherapists manage musculoskeletal disorders or the extent to which their practice aligns with international evidence-based recommendations. International studies have repeatedly demonstrated inconsistencies in guideline adherence, including high imaging utilisation, variable implementation of exercise-based rehabilitation, and widespread use of adjunct therapies [33,34,35,36,37,38,39]. Given the absence of comparable data in Cyprus, establishing current clinical practice patterns is essential for identifying knowledge-translation needs, informing national educational strategies, and situating Cypriot practice within the broader international landscape.

Therefore, the primary aim of this current study was to investigate the current physiotherapy management of RCRSP among Cypriot physiotherapists. The secondary aim was to compare how reported practice adheres to recommended patient management. The tertiary aim was to compare the management of Cypriot physiotherapists of RCRSP to that reported in other countries (the United Kingdom, Belgium, the Netherlands, Australia, France, Italy and Greece).

## 2. Materials and Methods

### 2.1. Study Design

The present study is a cross-sectional, anonymous online survey that gathered information from physiotherapists in Cyprus, exploring the management of RCRSP based on the STROBE guidelines [41] for cross-sectional studies and the CHERRIES (Checklist for Reporting Results of Internet E-Surveys) guidelines [42]. The design was adapted from previously published studies with the permission of the authors [36,37]. The protocol was approved by the Cyprus bioethics committee (ΕΕΒΚ ΕΠ 2024.01.172/2024-05-30), and all procedures were conducted in accordance with the Declaration of Helsinki.

### 2.2. Sampling and Data Collection

Data were collected online from June to July 2024. Based on previous surveys, a one-month period was established, recognising that most responses are typically received within the first one to two weeks [34]. Potential participants were invited to complete the survey through multiple sources. The survey link was distributed via the Cyprus Physiotherapy Association email newsletter on two occasions. Additional reminders were shared on social media, specifically within Cyprus-based physiotherapy Facebook groups. Furthermore, follow-up email invitations were sent to individuals from a contact list, one week after the initial email and again two weeks later, to reach additional potential respondents [43]. Participants’ informed consent for the survey was implied through voluntary completion without requiring a written consent form. Physiotherapists were excluded if they were not working in Cyprus. Only complete responses were included in the analysis. All responses were anonymous, and no personal data was collected. The system was configured to prevent multiple submissions from the same IP address.

### 2.3. Sample Size

No power calculation was performed for the current survey due to the exploratory nature of this cross-sectional study. Our approach was to obtain the maximum number of responses in a defined period. This approach is reflective of other similar surveys [34,35]. We aimed to recruit approximately 150 participants to ensure that the number of responses to this survey was comparable with previous surveys [34,44]. Given the smaller national workforce, the sample still provides a meaningful representation of physiotherapy practice in the country.

### 2.4. Survey Questionnaire

**Figure 1:**
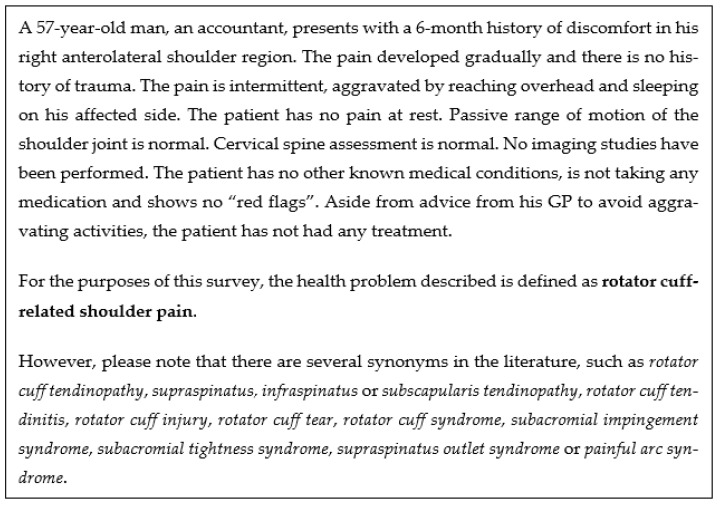
Clinical vignette.

The survey was originally designed by Bury and Littlewood [34] and then adapted by Smythe et al. [36]. The core structure and content of the survey and vignette were retained from the original work. With the authors’ permission, the survey of Smythe et al. [36] was translated into Greek and adapted to fit the Greek healthcare context (Supplementary File S1). The English version is included as Supplementary File S2. The survey was created with Microsoft Forms (Microsoft Corp., Redmond, WA, USA) and underwent cognitive interviewing with a convenience sample of two Greek physiotherapists to assess clarity and potential online operational issues [39]. Minor subsequent adjustments were made in response to feedback from the cognitive interviews. The survey consisted of 32 questions divided into three sections. The first section included demographic characteristics, such as age, gender, level of clinical experience, work setting (e.g., private practice), location (e.g., town), highest level of qualification, and whether they had a particular interest in shoulder pain (questions 1–10). The second section focused on clinical reasoning in RCRSP, explored through a clinical vignette that encapsulated a typical presentation of RCRSP, modified from the work of Bury and Littlewood [34] (Figure 1). Such vignettes are valid for reflecting on clinical practice and decision-making [45]. Specific open-ended questions were converted into a multiple-choice format to increase completion rates. The potential answers to the multiple-choice questions were derived from the original work of Smythe et al. [36] and Riera et al. [37], who performed content analysis to extract data from open-ended questions. Subsequent multiple-choice and open-ended questions explored treatment decision-making, including the frequency and duration of treatment related to the vignette (questions 11–23). The third and final section included additional multiple-choice questions (questions 24–32) designed to investigate specific practices of Cypriot physiotherapists regarding exercise prescription and education.

### 2.5. Determination of Recommended Care

Answers provided by participants were compared against relevant guidelines [6,18,25,27,35,46]. These guidelines were selected for their relevance to non-traumatic rotator cuff–related shoulder pain and their frequent use in previous international surveys, which allows methodological comparability. The specific recommendations, their Levels of Evidence (LoE), and the full synthesis used to benchmark participant responses as reported in the original guideline documents are summarised in Supplementary File S3.

### 2.6. Statistical Analysis

All survey data were exported from Microsoft Forms to Microsoft Excel 365 (Microsoft Corp., Redmond, WA, USA). The data from the closed-ended questions were entered into the IBM SPSS Statistics software (Version 29, IBM SPSS Inc., Chicago, IL, USA) for statistical analysis. First, descriptive statistics (frequency tables) were extracted, followed by the performance of: (a) Chi-square tests to examine the association between the variable *Surgery* and six variables (*Special interest in shoulder pain or rotator cuff-related pain, Work Setting, Level of post-graduate education, Age, Years working with RCRSP patients,* and *Years qualified*), (b) Chi-square tests to examine the association between the variable *Injection* and six variables (*Special interest in shoulder pain or rotator cuff related pain, Work setting, Level of education, Age, Years working with RCRSP patients,* and *Years qualified*), and (c) Binary logistic regression with the dependent variable *Imaging* and eight independent variables (*Surgery, Injection, Special interest in shoulder pain or rotator cuff related pain, Work setting, Level of post-graduate education, Age, Years working with RCRSP patients,* and *Years qualified*). The significance level for all analyses was set at 0.05.

For statistically significant associations identified in the Chi-square tests, Cramer’s V was calculated as the effect size measure, while in the binary logistic regression, Exp (b) was used as the effect size measure. All Chi-square tests met the assumptions of (a) Total sample size > 40, and (b) No more than 20% of the expected values being less than 5, with none less than 1 (no empty cells) [47,48]. Cramer’s V values were interpreted based on the following thresholds: 0–0.10 (Negligible association), 0.10–0.20 (Weak association), 0.20–0.40 (Moderate association), 0.40–0.60 (Relatively strong association), 0.60–0.80 (Strong association), and 0.80–1.00 (Very strong association) [49].

For the binary logistic regression, the following assumptions were checked and met: (a) The observations must be independent, (b) There must be no perfect multicollinearity among the independent variables, and (c) Continuous predictors must be linearly related to a transformed version of the outcome (no continuous variables were included in our model) [50]. The minimum sample size was determined based on the widely accepted criterion that suggests a minimum of 10 events per predictor variable to ensure reliable model estimates and avoid overfitting [51].

Open-ended answers were transcribed verbatim. Microsoft Excel 365 (Microsoft Corp.) was used to manage the qualitative data. A qualitative content analysis methodology was employed, allowing large amounts of data to be reduced into workable themes [52,53]. Two researchers identified units of meaning by reading each response and manually developing initial codes. These codes were then organised into initial categories based on the common topics of the open questions in physiotherapy management. The codes were further refined into categories, and a descriptive column was added to the Microsoft Excel spreadsheet. Additionally, the frequency of categorical descriptions was also analysed.

## 3. Results

### 3.1. Descriptive Statistics

A total of 143 physiotherapists accessed and completed the survey of the 1300 licensed physiotherapists in Cyprus (11% response rate). Table 1 presents the demographic characteristics of the respondents, reflecting the composition of the study sample. The majority of participants (75%) identified as male (n = 107/143), 79% expressed a specific interest in shoulder pain or RCRSP (n = 113/143), worked in the capital (57%, n = 82/143), 83% practised in private settings (n = 118/143), 57% had received post-graduate training in the form of seminar/1–2 days training course (n = 81/143), and 40% were qualified for >5 years (n = 57/143). Moreover, 46% of the respondents had been working with RCRSP patients for ≤5 years (n = 66/143), and 46% had been treating shoulder pain cases on average of 6–10 per month (n = 56/143). Regarding the respondents’ clinical interests, the majority (62%) primarily treated patients with musculoskeletal and other complaints (n = 89/143).

### 3.2. Care Recommendations for the Clinical Vignette

#### 3.2.1. Referrals

Most respondents did not recommend imaging (64%, n = 91/143). Among the minority that recommended imaging, the most recommended imaging modality was MRI (59%, n = 43/73), followed by X-ray (26%, n = 19/73) and ultrasound (15%, n = 11/73).

**Figure 2:**
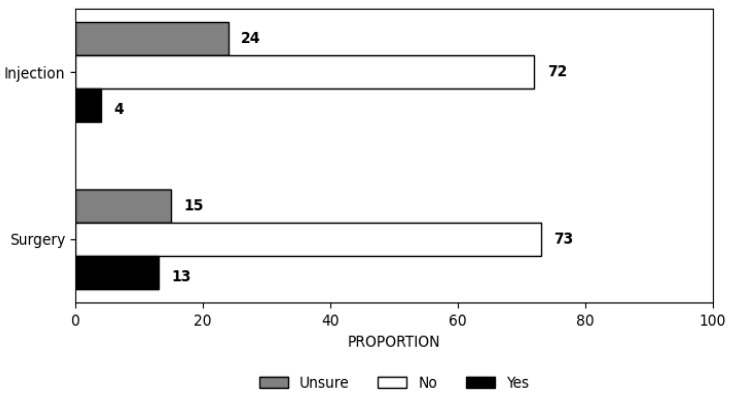
Proportion of physiotherapists recommending referral for the clinical vignette.

Most physiotherapists did not recommend a referral for an injection in the case presented (72%, n = 103/143). The remaining were either unsure (24%, n = 34/143) or would recommend injection (4%, n = 6/143). A small proportion of physiotherapists would refer for surgical opinion (12%, n = 17/143) or were unsure whether to refer (15%, n = 22/143) in reference to the clinical vignette (Figure 1), while most would not (73%, n = 104/143). Referral recommendations in response to the clinical vignette are shown in Figure 2.

Table 2 shows the variables entered into the χ^2^ tests and binary logistic regression models. Work setting, level of post-graduate education, age, years working with RCRSP patients, and years qualified were not associated with undergoing surgery or receiving an injection. Physiotherapists with a special interest in shoulder pain or rotator cuff-related pain (79%, n = 113/143) were more likely to recommend surgery (χ^2^ = 4.937, *p* = 0.026) and injection (χ^2^ = 9.143, *p* = 0.002). In the first case, the association is weak (Cramer’s V = 0.186), while in the second, it is moderate (Cramer’s V = 0.253) (Table 3).

The procedure modelled MRI as the response, treating No/X-Ray/Ultrasound as the reference category. The Omnibus test is statistically significant (*p* < 0.001). Cox & Snell R Square = 0.249 and Nagelkerke R Square = 0.374. Table 4 shows that only the Work setting and Surgery variables have a statistically significant effect on the model. In particular, Work setting increases the probability of MRI by 5 times compared to Private, while Yes/Unsure Surgery increases the probability of MRI by 7 times.

#### 3.2.2. Exercise Prescription

**Figure 3:**
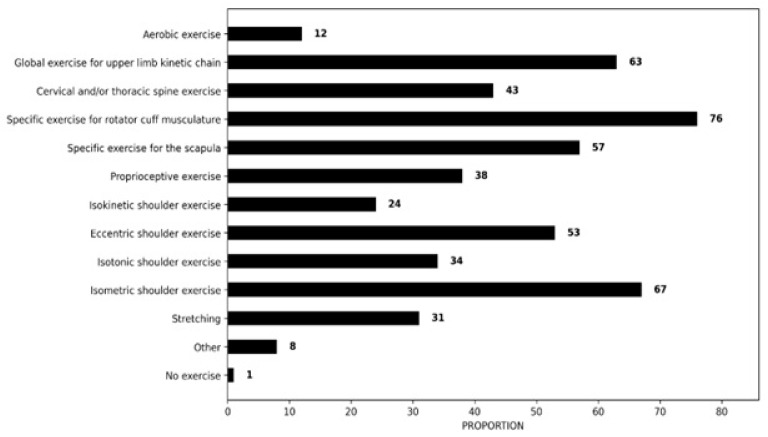
Proportion of recommended exercise strategies.

Figure 3 illustrates that physiotherapists recommend various strategies for RCRSP. Consistent with current treatment recommendations, 99% of respondents (n = 142/143) prescribed some form of exercise therapy in response to the case vignette shown in Figure 1. The most popular exercise included exercise for rotator cuff muscles (76%, n = 109/143), isometric shoulder exercise (67%, n = 96/143), global exercises for the entire upper limb kinetic chain (63%, n = 90/143), specific exercise for the scapula (57%, n = 82/143), eccentric shoulder exercise (53%, n = 76/143), cervical and/or thoracic spine exercise (43%, n = 62/143) Less than 40% of participants recommended proprioceptive (38%, n = 55/143) or isotonic exercise (34%, n = 49/143) or stretching exercise (31%, n = 45/143). Isokinetic shoulder exercise (24%, n = 34/143), aerobic exercise (12%, n = 17/143) and other (8%, n = 12/143) were the least frequent responses.

#### 3.2.3. Education

All participants stated that they would provide some form of education for RCRSP management to the patient described in the clinical scenario in line with guideline recommendations, as shown in Table 5. This included activity modification in response to pain (81%, n = 116/143), providing information about the pathology of RCRSP (78%, n = 111/143) and education about risk factors (76%, n = 109/143). 72% (n = 103/143) of physiotherapists would provide education regarding recommended physiotherapy management, while 65% (n = 93/143) of the physiotherapists surveyed would provide education on factors that influence pain and explore the relationship between pathology and pain (63%, n = 90/143). A minority of physiotherapists reported discussing the role of educating patients on the timeframe or indication for imaging (38%, n = 55/143), surgery (16%, n = 23/143), injection (13%, n = 18/143) and other (4%, n = 6/143).

#### 3.2.4. Adjunctive Treatment

**Figure 4:**
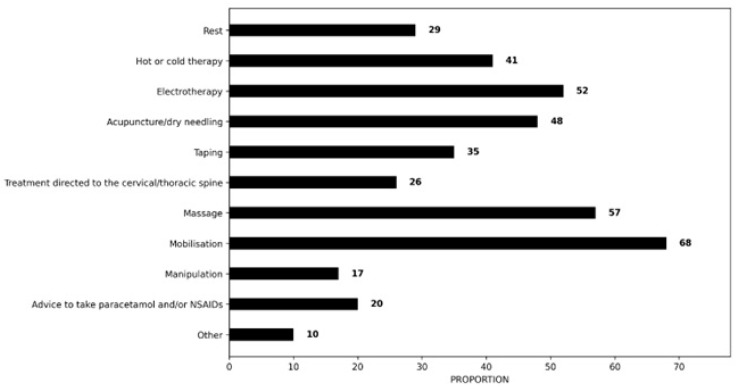
Proportion of recommended adjunctive treatment.

Regarding adjunctive treatment, most physiotherapists would provide mobilisation (68%, n = 97/143), massage (57%, n = 81/143) and electrotherapy (52%, n = 74/143). Less than half (48%, n = 68/143) would perform acupuncture or dry needling, 41% (n = 58/143) would use hot or cold therapy, and 35% (n = 50/143) would perform another method. The least recommended approaches included rest (29%, n = 42/143), treatment directed towards the cervical or thoracic spine (26%, n = 37/143), the use of paracetamol and oral anti-inflammatories (20% n = 29/143), manipulation (17%, n = 25/143) and other interventions (10%, n = 14/143) (Figure 4).

#### 3.2.5. Information Formats

Physiotherapists predominantly rely on written or printed information (65%, n = 93/143), followed by video recordings (57%, n = 82/143) and verbal information (53%, n = 76/143). Only a minority would provide links to online videos or websites (28%, n = 40/143).

#### 3.2.6. Management Frequency and Duration

Almost all physiotherapists would recommend seeing patients with RCRSP either weekly (62%, n = 88/143) or fortnightly (30%, n = 43/143) to review or adjust exercises. Less common frequencies included reviews every three weeks (3%, n = 4/143) or not at all since the original prescription (3%, n = 4/143) and at least once a month or more (2%, n = 3/143). Only one respondent (1%, n = 1/143) stated they would not modify exercises after the initial prescription.

**Figure 5:**
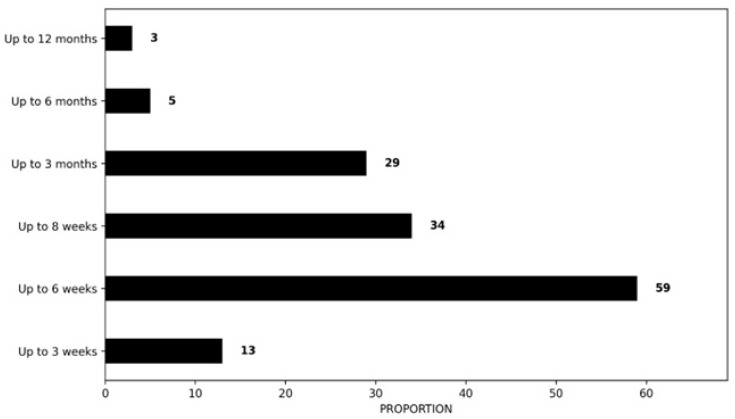
Proportion of expected treatment duration.

Regarding the duration of treatment, 41% (n = 59/143) of the respondents would expect to treat RCRSP patients for 6 months or longer, whereas 24% (n = 34/143) would expect a treatment period of up to 8 weeks. A smaller percentage, 20% (n = 29/143), would expect to see patients up to 3 months, 9% (n = 13/13) up to 3 weeks, 3% (n = 5/143) up to 6 months and 2% (n = 3/143) up to 12 months (Figure 5).

#### 3.2.7. Guidelines for Exercise Prescription

**Figure 6:**
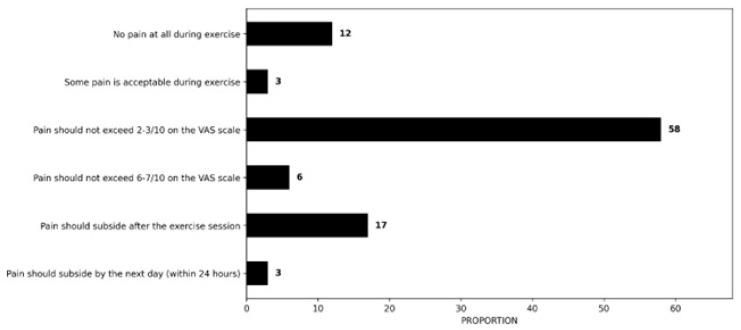
Pain management guidelines for exercise prescription.

When it comes to exercise guidance, most respondents indicated that experiencing pain during exercise is acceptable, provided it does not exceed 2–3/10 on the Visual Analogue Scale (VAS) (58%, n = 83/143). A smaller proportion, 17% (n = 24/143), reported that pain should subside after the exercise session, while 12% (n = 17/143) reported that no pain at all should be experienced during exercise. Additionally, 6% (n = 9/143) reported pain should not exceed 6–7/10 on the VAS scale, whereas 3% (n = 5/143) believed that some pain is acceptable during exercise or pain should subside by the next day (within 24 h) (Figure 6).

#### 3.2.8. Guidelines for Prescribing Exercise Load and Resistance Intensity

Most respondents (43%, n = 61/143) reported load intensity being based on patient symptoms (e.g., a load that does not cause pain greater than 4–5/10 on the VAS scale), while 29% (n = 42/143) start with a low load (e.g., a dumbbell of 1–2 kg). Additionally, 10% (n = 15/143) determine the load based on the maximum load to maintain movement quality and avoid compensation or 10% (n = 14/143) based it on the training goal (e.g., strength, hypertrophy, or endurance), 4% (n = 6/143) used the level of fatigue induced (e.g., a load that leads to significant fatigue at 12 repetitions until failure) and 3% (n = 5/143) reported load being based on specific repetition ranges, e.g., 60–70% of 1RM [one repetition maximum] (Table 6).

#### 3.2.9. Guidelines for Prescribing Repetitions and Sets in Exercise Recommendations

Clinical considerations concerning repetitions and sets varied. Most respondents (77%, n = 110/143) suggested that sets and repetitions should be adapted to the patient’s symptoms and irritability. A smaller proportion 13% (n = 19/143) based their recommendations on treatment goal (e.g., 3 × 45-sec hold for isometric exercises, 3 sets × 12 repetitions for isotonic exercises), 6% (n = 9/143) cited specific repetitions and sets for everyone ranging (e.g., 3 sets of 12 repetitions), while 3% (n = 5/143) cited other methods (Table 7).

#### 3.2.10. Guidelines for Prescribing Exercise Frequency

Most respondents suggested daily exercise (31%, n = 45/143) or prescribed a frequency based on the patient’s symptoms (30%, n = 43/143). A smaller proportion, 14% (20/143), recommended performing the exercises 3–5 times per day or several times per week (3–5 times per week). Few would prescribe frequency depending on the treatment goal (e.g., strengthening, hypertrophy, etc.) (9%, n = 13/143). No one based on fatigue levels, and 1% (n = 2/143) answered other (Table 8).

#### 3.2.11. Guidelines for Exercise Progression and Regression in Load Adjustment

Exercise intensity adjustments varied among physiotherapists, with most respondents (50%, n = 71/143) recommending modifications by either increasing or decreasing the load. A significant proportion (26%, n = 37/143) suggested adjusting the number of sets/repetitions, while 15% (n = 22/143) focused on adjusting the range of motion. A smaller proportion (9%, n = 13/143) provided other methods (Table 9).

### 3.3. Qualitative Results

Responses to open-ended questions were analysed using content analysis. The focus was on clinical indications for imaging, injection, or surgical intervention.

#### 3.3.1. Regarding the Clinical Indications for Performing an X-Ray

Most respondents (86%, n = 123/143) reported that X-ray imaging was unnecessary in the given clinical scenario. Open-ended responses were categorised into three thematic groups: structural and anatomical abnormalities, focusing on acromion morphology, osteophytes, and subacromial space reduction; pathological conditions and differential diagnosis, emphasising the identification of specific conditions such as impingement syndrome, arthritis, and calcific tendinitis; and diagnostic decision-making and clinical judgment, providing insights into when and why diagnostic evaluations are pursued based on clinical symptoms and the persistence of pain.

A common response to structural and anatomical abnormalities was: *“To identify structural abnormalities such as osteophytes, acromion type variations, and reduction of subacromial space that may contribute to mechanical impingement.”* This reflects concerns related to bony changes (e.g., type II/III acromion, osteophytes) and their potential role in impingement or reduction of the subacromial space, which may affect shoulder function. A common response for pathological conditions and differential diagnosis was: *“To investigate specific shoulder pathologies, including calcific tendinitis, impingement syndrome, and acromioclavicular arthritis, particularly in cases presenting with persistent pain and reduced range of motion.”* This highlights the focus on diagnosing common shoulder conditions that could explain pain, limited active range of motion, and functional impairments. Lastly, a common response under diagnostic decision-making and clinical judgment was: *“To support clinical decision-making when persistent symptoms do not improve with conservative management, typically after several months, or when the diagnosis remains unclear.”* This captures the rationale for initiating diagnostic imaging or further evaluations based on clinical judgment, symptom persistence, and inadequate response to treatment.

#### 3.3.2. Regarding the Clinical Indications for Performing a US Scan

Most respondents (92%, n = 132/143) indicated that US imaging was unnecessary in the given clinical scenario. The open-ended responses were categorised into three thematic groups. The first theme, tendon pathologies and soft tissue assessment, focused on detecting tendon-related issues such as tears, inflammation, and calcifications and evaluating the subacromial space and potential impingement mechanisms. A common response within this theme was: *“To assess soft tissue structures, including the presence of tendon tears, inflammation, and calcifications, as well as to evaluate the subacromial space and potential impingement mechanisms.”* The second theme, differential diagnosis and exclusion of serious pathologies, highlighted the role of imaging in supporting differential diagnoses, particularly for identifying calcifications and ruling out more serious conditions such as carcinoma, especially in cases with atypical pain patterns or red flags. A representative response for this theme was: *“To support differential diagnosis by identifying calcifications and ruling out serious conditions such as carcinoma, especially in cases with atypical pain patterns or red flags.”* The final theme, diagnostic effectiveness and imaging preferences, reflected the perceived superiority of US as an imaging modality for shoulder pathologies due to its effectiveness in assessing soft tissue structures and allowing dynamic evaluation. A common response in this category was: *“To utilise US as a preferred imaging modality due to its effectiveness in assessing soft tissue structures and dynamic shoulder evaluation.”*

#### 3.3.3. Regarding the Clinical Indications for Performing an MRI Scan

Most respondents (74%, n = 106/143) indicated that MRI was unnecessary in the given clinical scenario. The open-ended responses were categorised into three thematic groups. The first theme, assessment of soft tissue pathologies and structural abnormalities, focused on evaluating soft tissue structures, particularly the rotator cuff, tendons, and labrum, to identify potential pathologies such as tendon tears, tendinopathies, inflammation, and impingement syndromes. Responses emphasised the importance of understanding the extent of tissue damage, differentiating between types of injuries, and assessing anatomical abnormalities that may contribute to symptoms. A common response within this theme was: *“To assess soft tissue structures, including tendons and labrum, for potential tears, tendinopathies, inflammation, and anatomical abnormalities, guiding both diagnosis and treatment planning.”*

The second theme, diagnostic decision-making based on clinical presentation, captured responses related to clinical reasoning and criteria for determining when imaging is necessary. This was particularly evident in cases of persistent pain, lack of response to conservative treatment, or when clinical findings were ambiguous. Factors influencing decision-making included the chronicity of symptoms, pain patterns, and passive range of motion assessments. A representative common answer for this theme was: *“To support clinical decision-making when persistent symptoms do not improve with conservative management, particularly in cases of chronic pain, unresponsive symptoms, or inconclusive clinical findings.”*

The final theme, imaging preferences and perceived diagnostic effectiveness, reflected the perceived superiority of certain imaging modalities, particularly MRI, due to its effectiveness in evaluating soft tissue structures, absence of ionising radiation, and ability to provide comprehensive diagnostic information. Respondents highlighted the role of MRI in confirming diagnoses, especially for soft tissue involvement and differential diagnosis. A common response within this group was: *“To utilise MRI as the preferred imaging modality due to its effectiveness in providing detailed assessments of soft tissue structures, without ionising radiation, and its role in guiding targeted treatment strategies.”*

#### 3.3.4. Regarding the Clinical Indication for Injectable Therapy

Based on the clinical scenario, 95% of physiotherapists (n = 136/143) stated that injectable therapy was unnecessary. The open-ended responses were categorised into three thematic groups. The first theme, injectable therapy as a last resort after conservative management failure, reflected the view that injections should only be considered when conservative treatments, such as physiotherapy, exercise, and medication, have not led to significant clinical improvement. Respondents emphasised the importance of exhausting all non-invasive options before resorting to injections. A common response within this theme was: *“Only if physiotherapy, exercise, and medication did not improve the patient’s clinical condition, an injection would be considered a possible solution.”*

The second theme, injectable therapy in cases of persistent or unmanageable pain, highlighted situations where persistent pain, especially pain interfering with daily activities or rehabilitation, might warrant the consideration of injections. This was often linked to cases where pain was resistant to conventional therapies or potentially associated with inflammatory processes that could not be effectively managed through medication alone. A representative common response for this theme was: *“Persistent pain that may be due to an inflammatory process and cannot be managed with medication may warrant the consideration of an injection.”*

The third theme, injectable therapy based on patient-related factors, included responses suggesting that injections might be necessary for specific patient-related circumstances, such as non-compliance with rehabilitation instructions or a limited ability to engage in exercises due to severe pain. A common response within this group was: *“For a patient who does not comply with my instructions, an injection might be considered to facilitate pain reduction and improve participation in rehabilitation.”*

#### 3.3.5. Regarding the Clinical Indication for Surgery

Based on the clinical scenario, 88% of physiotherapists (n = 126/143) stated surgery was unnecessary. The open-ended responses were categorised into three thematic groups. The first theme, surgery as a last resort following the failure of conservative management, emphasised that surgery should only be considered after exhausting conservative treatments such as physiotherapy, exercise, and medication, notably when these interventions have not yielded satisfactory results. This perspective underscores the importance of prioritising non-invasive treatments before escalating to more invasive procedures. A common response within this theme was: *“Only after exhausting conservative treatment without results would surgery be considered.”* The second theme, surgery in cases of confirmed structural damage or severe pathology, focused on situations where significant structural issues, such as tendon ruptures, full-thickness rotator cuff tears, or advanced degenerative changes, were present. Respondents indicated that surgery may be appropriate when structural pathology is confirmed, particularly through diagnostic imaging, and is associated with persistent symptoms or functional limitation. A representative response for this theme was: *“Surgery may be considered in cases of confirmed structural damage, such as a severe rotator cuff tear or tendon rupture.”* The third theme, surgery for severe, unmanageable, or chronic pain, addressed cases where persistent, severe pain significantly impacts daily functioning, sleep, or quality of life, and is unresponsive to standard treatments. This group also included situations where severe, persistent pain that substantially affected daily functioning and quality of life despite comprehensive conservative management. A common response within this group was: *“Surgery might be appropriate for managing severe, chronic pain that does not improve despite multiple conservative treatments, especially when pain significantly affects daily activities and sleep.”*

## 4. Discussion

This study examined how physiotherapists in Cyprus adhere to internationally recommended management strategies for RCRSP. Almost all respondents recommended some form of education and exercise as management, minimal use of imaging, injection, and surgery, indicating consistency with guideline-recommended care. However, some discrepancies exist, particularly in exercise prescription and adjunct therapies. Findings are comparable to those found internationally in nations such as the United Kingdom, Belgium/the Netherlands, Australia, Italy and Greece; Cypriot physiotherapists exhibited variability in the prevalence and methodology of guideline-congruent management. Such variations may be attributed to local educational frameworks, healthcare system characteristics, and patient expectations.

### 4.1. Imaging, Injection, Surgery

The study findings on Cypriot physiotherapists’ referral practices for imaging, injections, and surgery reveal a strong inclination towards conservative management strategies, consistent with international guidelines that advocate for non-invasive approaches in the absence of red flags [16,25]. Imaging is indicated if a 4–6 week trial of active treatment fails, and both injection therapy and surgical consultation should only be considered after 12 weeks of conservative management [16,18,25]. Consistent with guideline recommendations, most respondents (64%, n = 91/143) would not recommend imaging for the patient in the case vignette and would not consider a surgical opinion (73%, n = 104/143). This is encouraging, as premature imaging and early surgical referral have been associated with increased healthcare costs, patient anxiety, and unnecessary interventions in musculoskeletal disorders [54]. Over-reliance on imaging may reinforce pathoanatomical beliefs in patients, potentially undermining self-management and recovery [55].

Injection was not recommended by most respondents (72%, n = 103/143) in line with guideline recommendations. The remaining respondents were uncertain (24%, n = 34/143), and only a minority would recommend injection (4%, n = 6/143). However, it is noteworthy that a subset of physiotherapists exhibited deviations from these guidelines, indicating variability in practice that may be influenced by personal interest or specialisation in shoulder-related conditions.

A statistically significant association was observed between special interest in shoulder pain or rotator cuff-related pain, surgery, and injection (*p* = 0.026 and *p* = 0.002, respectively). The association with surgery was weak (Cramer’s V = 0.186), while the association with injection was moderate (Cramer’s V = 0.253). This finding suggests that physiotherapists with a special interest in shoulder pain or rotator cuff-related pain were more likely to recommend surgical interventions and injections. This finding is counterintuitive, as one might expect that physiotherapists with a special interest in shoulder conditions would demonstrate stronger alignment with evidence-based guidelines. However, it is important to note that this “special interest” was self-reported and may not equate to formal training or advanced clinical expertise. Previous literature suggests that self-perceived expertise does not always translate into guideline adherence [38,39]. It is also possible that increased interest may lead to a more biomedical interpretation of pathology, influencing decisions towards diagnostic imaging or interventional referrals. Studies have also shown that clinicians with a strong interest in a specific area may also be more confident in deviating from guidelines based on personal experience, perceived clinical complexity, or outdated knowledge [38,56]. This highlights the need for continuous professional development and critical appraisal skills to ensure that clinical interests are aligned with current evidence-based practice.

Despite the noted associations, most physiotherapists (76%) preferred basic imaging techniques, reserving advanced imaging such as MRI for specific cases, particularly when considering surgery or injections. The study found that MRI use was significantly associated with recommendations for surgery (“Yes/Unsure”), with those recommending MRI being almost seven times more likely to do so (Exp(B) = 6.944, *p* < 0.001), suggesting a reliance on advanced imaging to inform surgical decisions. This aligns with the established role of MRI in confirming surgical indications, such as assessing rotator cuff integrity or ruling out other complex shoulder pathologies. The reliance on MRI in these cases reflects its utility in enhancing diagnostic accuracy and guiding invasive interventions, consistent with global practices [57,58,59]. Nevertheless, this tendency towards advanced imaging raises concerns about the potential for overdiagnosis and overtreatment, as highlighted by previous studies linking premature diagnostic imaging to adverse outcomes, including the nocebo effect [60,61,62]. For example, patients with shoulder pain may misinterpret imaging findings, such as tendon tears, as necessitating surgical intervention, even when conservative management could suffice [63,64]. Therefore, physiotherapists must contextualise imaging results accurately and align them with evidence-based care principles to mitigate misconceptions.

Physiotherapists working in non-private settings were five times more likely to use MRI than those in private practice (Exp(B) = 5.049, *p* = 0.014). This suggests that access to advanced imaging modalities may be more readily available in public or institutional work settings, where structured diagnostic protocols often govern patient care [65,66]. Additionally, economic and administrative factors, such as equipment availability, reimbursement incentives, or pressure to justify care pathways, can influence clinical decisions, potentially leading to the overuse of imaging [58,67]. These system-level considerations may partly explain why physiotherapists in non-private settings were significantly more likely to recommend MRI, highlighting the importance of aligning institutional protocols with up-to-date clinical guidelines. In contrast, private practice settings may prioritise cost-effectiveness and favour conservative approaches, aligning with international recommendations to reserve MRI for cases where it directly influences management decisions [67,68].

In terms of injection practices, the study found that 28% of physiotherapists were either uncertain about or recommended injections despite guidelines discouraging their routine use without clear indications [16,25]. The long-term efficacy of corticosteroid injections remains contentious, with research indicating potential risks such as persistent pain and tendon degradation and an increased likelihood of injury recurrence [69,70,71]. This underscores the necessity for ongoing professional development to keep patients informed about the adverse effects and limited benefits of such interventions. Furthermore, only 27% of participants suggested surgery, reinforcing the conservative approach observed among the majority. However, the association between MRI use and surgical recommendations suggests a reliance on advanced diagnostics that may inadvertently influence decision-making towards more invasive options.

Compared to other countries, Cypriot physiotherapists were consistent in their recommendations for injection management, considering guideline recommendations (72%), similar to France and Australia, with over 70% [36,37]. The referral rate for imaging by Cypriot physiotherapists (36%, n = 52/143) is higher than those in Australia (6.4%), the United Kingdom (9.0%), France (12.6%), and Belgium/the Netherlands (31.0%), but lower than in Italy (42.0%) and Greece (44%) [34,35,36,37,38,39]. No indication for surgery was stated in 73% (n = 104/143) of Cypriot physiotherapists compared to Greece (74%), Australia (90.0%), France (89.3%), Italy (62.0%), Belgium/the Netherlands (70.0%) [35,36,37,38,39]. This pattern may be due to different practice standards; in practices in countries like France, Australia, the Netherlands, and the United Kingdom, physiotherapists have expanded their scope of practice as first-contact practitioners, necessitating decision-making regarding referral for interventionalist care [72,73,74,75,76] compared to Belgium, Italy, Greece, and Cyprus [56].

### 4.2. Education

Education is recommended as an essential component of RCRSP management; however, guidelines provide limited detail on specific educational topics and delivery methods beyond advice related to activity modification and risk factors [16,18,25]. This is evident in the education approaches adopted by practitioners. Consistent with guideline recommendations, all Cypriot physiotherapists reported providing some form of education, although there was variability in the specific topics covered. Cypriot physiotherapists were more likely to recommend education on activity modification (81%, n = 116/143) compared to physiotherapists in France (69.9%), but this was similar to rates in Australia (85.3%), Greece (83%), and Belgium/the Netherlands (80.0%) [35,36,37,39]. They were also less likely to recommend education concerning the timing and indication for imaging (38%, n = 55/143), injection (13%, n = 18/143) and surgery (16%, n = 23/143) compared to Australian physiotherapists (50.4% 35.7% and 25.1%, respectively) [36], but more likely than French (14.6%, 7.8% and 6.3%, respectively) [37] and Greek physiotherapists (28%, 9% and 12%, respectively [39]. This may be because, in Australia, physiotherapists are first-contact practitioners [72], meaning patients can access their services without a referral from another medical professional. In contrast, in Cyprus, Greece, and France, such referrals are typically required [73]. However, there was considerable variation in the educational strategies used, highlighting a lack of consistency. This emphasises the need for more precise guidelines to reduce this variability and promote uniformity in educational approaches.

### 4.3. Exercise Prescription

The physiotherapeutic management of RCRSP is characterised by a high prevalence of exercise prescription among Cypriot practitioners, with 99% (n = 142/143) of surveyed physiotherapists incorporating exercise as an integral component of their treatment protocols. This practice is in line with guideline recommendations, as evidenced by responses to the clinical vignette [16,18,25,46]. However, this widespread adoption is accompanied by significant variability in the specific practices used to deliver exercise, such as dosage, progression, type of exercise, etc. This variability can be attributed to the extensive range of exercise trials and the inherent heterogeneity of interventions documented in the literature [21,77] and the lack of evidence demonstrating the superiority of any specific type of exercise over another [21].

The survey demonstrated that a significant proportion of physiotherapists prioritised exercise interventions targeting the rotator cuff musculature (76%, n = 109/143), isometric shoulder exercise (67%, n = 96/143), global exercises addressing the entire upper limb kinetic chain (63%, n = 90/143) and scapula-specific exercises (57%, n = 82/143). These exercises are consistent with evidence-based recommendations in the current literature [24,26,31,78,79] and reflect similar findings in France, Australia, Belgium/The Netherlands, Italy and Greece [33,34,35,36]. Regarding contraction type, more respondents recommended isometric exercise (67%, n = 96/143) than eccentric (53%, n = 76/143) or isotonic (34%, n = 49/143). This was similar to findings in Australia, France, Italy, and Greece that recommended isometric exercise despite the limited evidence supporting its effectiveness [21].

Patient symptoms were the primary factor influencing decisions to consider or adjust exercise parameters. Pain was consistently a major factor in determining exercise parameters. However, the specific symptom thresholds used to guide these decisions varied considerably. Most respondents (58%, n = 83/143), when explaining, identified pain scale values of up to 2–3/10 as acceptable, while 17% (n = 24/143) reported pain should subside after the exercise session. This is consistent with research suggesting that moderate pain levels, up to 5/10, may offer potential benefits compared to pain-free exercise in the management of RCRSP [80]. Additionally, a consensus study suggests that experiencing mild to moderate pain (NRS/VAS ≤ 4/10) during exercise is acceptable as long as it returns to baseline levels within 12 h [81]. However, some experts argue that exercise should not trigger patient-specific pain [39]. A systematic review and meta-analysis on chronic musculoskeletal pain found that “painful” exercise provided significant short-term benefits compared to “pain-free” exercise [82]. The potential effects of exercising with pain have been partly attributed to the modulation of the nociceptive-inhibitory system. Additionally, it has been suggested that exercising at higher loads, often associated with increased pain, may promote greater tissue adaptation and accelerate recovery [83].

Recommendations regarding exercise parameters varied considerably, highlighting again the heterogeneity of exercise protocols in the literature [26,78]. There was notable variation in recommendations related to exercise load, sets, repetitions, and frequency. Most respondents (43%, n = 61/143) reported determining load intensity based on patient symptoms (e.g., selecting a load that does not provoke pain exceeding 4–5/10 on the VAS scale), while 29% (n = 42/143) preferred starting with a low load (e.g., a 1–2 kg dumbbell). The majority of respondents (77%, n = 110/143) suggested that sets and repetitions should be adjusted according to the patient’s symptoms and irritability. Regarding exercise frequency, most physiotherapists recommended either daily exercise (31%, n = 45/143) or tailoring the frequency based on the patient’s symptoms (30%, n = 43/143). Overall, physiotherapists consistently highlighted the importance of progressively increasing exercise difficulty by modifying load or other dosage parameters, such as sets and repetitions. This approach aligns with current evidence and clinical guidelines [16,25].

### 4.4. Treatment Timeline

Almost all physiotherapists would recommend seeing patients with RCRSP either weekly (62%, n = 88/143) or fortnightly (30%, n = 43/143) to review or adjust exercises. A frequency of one therapy session per week was also frequently recommended in France (70%) [37], Australia (49.8%) [36] and Greece (55%) [39], consistent with expert consensus [83].

Regarding treatment frequency, 41% (n = 59/143) of the respondents would expect to treat RCRSP patients for 6 months or longer, whereas 24% (n = 34/143) would expect a treatment period of up to 8 weeks, results similar to those of Greek physiotherapists, which contradict the current guidelines that exercise programs should be undertaken for at least 12 weeks [16,25,27,46]. This diversity was also observed in the comparative countries [33,35,36,37,39]. Findings from France suggested that 84.0% of respondents would expect to see a RCRSP patient for up to 12 weeks of treatment, 60.2% in Australia, 50% in the United Kingdom, 38.8% in Belgium/the Netherlands, and 12% in Greece.

The need for further investigation into the dosage parameters for exercise management in RCRSP and the clinical reasoning physiotherapists employ when prescribing exercise is evident. As previously noted, the existing literature demonstrates significant variability in specific exercise prescriptions, as indicated by the findings of this study. Furthermore, the absence of a unified approach to exercise treatment for RCRSP may be attributed to the diverse clinical presentations of pain and dysfunction, which necessitate an individualised adjustment to exercise management. In this context, clinical reasoning plays a crucial role, enabling physiotherapists to effectively customise treatment strategies to meet each patient’s unique needs, thereby enhancing therapeutic outcomes [84,85].

### 4.5. Adjunctive Treatment

In addition to education and exercise therapy, respondents selected a range of treatment methods, varying in type and frequency. Most physiotherapists would provide some form of manual therapy [mobilisation (68%, n = 97/143)] based on the clinical scenario. This is consistent with guideline recommendations demonstrating short-term effects for manual therapy in combination with active exercise therapy in terms of pain reduction [16,18,25]. The findings are comparable to those of French (70%), Australian (62%) and Greek (67%) physiotherapists [36,37,39]. However, it is important to note that more recent research has raised concerns about the clinical relevance of incorporating manual therapy techniques in the management of RCRSP [86,87]. Recent evidence also suggests that cervical or thoracic spinal joint manipulation does not improve short-term pain intensity or function in persons with painful shoulder [88].

The findings of this survey highlight the use of adjunctive treatment interventions with limited (electrotherapy) or inconclusive (acupuncture, taping, massage) clinical value [24,25,26]. Despite evidence indicating that massage and electrotherapy provide no therapeutic benefit in the management of RCRSP, a considerable proportion of Cypriot physiotherapists reported their use in response to the clinical vignette (57%, n= 81/143 and 52%, n = 74/143, respectively), a pattern comparable to Greek physiotherapists (58% and 56%, respectively). This contrasts markedly with lower usage rates of electrotherapy observed among Australian (11.2%), French (12.1%), Belgian/Dutch (4%), and British (1%) physiotherapists [33,35,36,37], suggesting potential variability in clinical practice patterns across different healthcare contexts. Furthermore, the consequences of employing ineffective treatment modalities extend beyond simple inefficiency; they may significantly compromise the effectiveness of conservative management approaches, contribute to prolonged symptoms, and elevate the risk of patients requiring more invasive interventions, including imaging, injections, or surgical procedures, but also a cost-efficacy issue using ineffective treatments. This is particularly concerning in the context of shoulder pain management, where clinical decision-making should be firmly grounded in evidence-based practices that optimise patient outcomes while ensuring the judicious use of healthcare resources [24,25,26].

### 4.6. Limitations

This study has several limitations that should be acknowledged. Firstly, using convenience sampling introduces a risk of sampling bias [89]. Invitation to survey participation was through social media platforms, e-mail distribution lists and physiotherapy associations. This approach likely excludes physiotherapists who are less active on these platforms and will not have had the opportunity to respond. Due to how the survey was distributed, it is impossible to determine how many individuals viewed the advertisement versus those who completed it. Therefore, the findings may not accurately reflect the broader population of physiotherapists in Cyprus. Additionally, the reliance on self-reported data introduces the potential for response bias, where participants may provide answers influenced by social desirability or personal beliefs, potentially influencing the results [90]. Furthermore, the total number of respondents (n = 143), although lower than in similar studies conducted in larger countries, represents approximately 11% of all registered physiotherapists in Cyprus. Considering the smaller size of the national workforce, the sample may still offer a reasonably representative snapshot of current clinical practice. Additionally, the relatively small sample size, along with the limited expertise (36% of respondents) and experience (40% with ≤5 years of practice) among a significant portion of participants, may further constrain the robustness and applicability of the study’s conclusions.

Another significant limitation is the survey methodology. The online survey was developed using a translated and adapted questionnaire based on existing literature; however, its reliability and validity have not yet been evaluated. The language and cultural adaptation of the survey instrument pose additional limitations. While the questionnaire was translated into Greek and modified to suit the local context, the adequacy of the translation process and the effectiveness of the cultural adaptation are not fully assured, raising concerns about potential misinterpretations or cultural biases. Additionally, the Greek-translated version of the questionnaire did not undergo formal reliability testing (e.g., internal consistency or test–retest analysis), which should be considered when interpreting the findings.

Evidence indicates that vignette studies are valid, reliable, cost-effective, and practical for investigating clinical practice [42,91,92]. Vignette-based study designs offer valuable insights into clinical decision-making processes, facilitating the evaluation of care quality and adherence to guideline standards in a structured, reproducible manner [91]. Careful development of the case vignette, grounded in current research evidence, helps minimise the risk of bias [93]. Additionally, this vignette has undergone continuous updates, adaptations, and multiple pilot tests as part of international comparative studies [91]. The depiction of a typical patient presenting with symptoms and functional impairments related to rotator cuff pathology was adapted from Smythe et al. [36] and translated into Greek [39].

### 4.7. Future Research

The survey findings support the need for more high-quality studies to identify barriers to guideline implementation and evaluate strategies to enhance knowledge translation and clinical practice in Cyprus and similar healthcare settings.

## 5. Conclusions

Most physiotherapists in Cyprus treat patients with RCRSP in strong alignment with international clinical guidelines, particularly in the widespread adoption of education and exercise as primary management strategies. Cypriot physiotherapists demonstrated a preference for conservative approaches, with minimal reliance on imaging, injections, or surgical referrals. However, notable variability exists in exercise prescription parameters, adjunctive therapy use, and referral practices, suggesting inconsistencies in guideline adherence. The frequent use of adjunctive treatments with limited or unclear clinical value, such as electrotherapy and massage, highlights the need for continuous professional development to bridge the evidence–practice gap. Additionally, factors such as personal clinical interest, work settings, and years of experience influence decision-making, underscoring the complexity of clinical reasoning in practice. Overall, while Cypriot physiotherapists largely adhere to recommended practices, areas for improvement include optimising exercise prescription consistency, reducing the use of low-value interventions, and reinforcing evidence-based decision-making.

## Figures and Tables

**Table 1:** Respondents’ demographic characteristics (n = 143). Variables reflect the composition of the study sample.

Age	Frequency (Absolute)	Frequency in %
18–24	9	6%
25–34	70	49%
35–44	39	27%
45–54	19	13%
55–64	4	3%
65–74	2	1%
**Gender**		
Male	107	75%
Female	34	24%
Prefer not to answer	2	1%
**Location**		
Nicosia	82	57
Limassol	29	20
Larnaca	18	13
Paphos	7	5
Famagusta	7	5
**Years qualified**		
≤5 years	57	40%
6–10 years	28	20%
11–15 years	23	16%
16–20 years	13	9%
21–25 years	14	10%
26–30 years	2	1%
31 years	6	4%
**Years working with RCRSP patients**		
≤5	66	46%
6–10	30	21%
11–15	19	13%
16–20	13	9%
21–25	8	6%
26–30	2	1%
≥31	5	3%
**Average number of shoulder pain cases treated per month**		
≤5	49	34%
6–10	56	39%
11–20	25	17%
21–30	9	6%
>30	4	3%
**Work setting**		
Private practice	118	83%
Rehabilitation centre	1	1%
Residential care facility	1	1%
Hospital	8	6%
Academic	4	3%
Other	11	8%
**Level of post-graduate education**		
Seminar/1–2 days training course	81	57%
Master’s Degree	41	29%
None	16	11%
PhD	5	3%
**Special interest in shoulder pain or RCRSP**		
Yes	113	79%
No	30	21%
**Area of expertise**		
Musculoskeletal and other	89	62%
Only musculoskeletal	47	33%
Other	3	2%
Not working in a clinical setting	4	3%

*Abbreviation:* RCRSP = Rotator cuff-related shoulder pain.

**Table 2:** Variables included in the statistical analyses. These variables were used in chi-square tests and binary logistic regression models.

Variable	Categories	Frequency (n)	Percentage (%)
Imaging	No/X-Ray/Ultrasound	109	76
	MRI	34	24
Injection	Yes/Unsure	40	28
	No	103	72
Surgery	Yes/Unsure	39	27
	No	104	73
Work setting	Other	25	17
	Private	118	83
Level of post-graduate education	PhD/MSc	46	32
	Seminar/None	97	68
Age	18–34	79	55
	35–44	39	27
	>44	25	17
Years working with RCRSP patients	≤5	66	46
	6–10	30	21
	11–15	19	13
	16–20	13	9
	>20	15	11
Years qualified	1–5	57	40
	6–10	28	20
	11–15	23	16
	16–20	13	9
	>20	22	15
Special interest in shoulder pain or rotator cuff-related pain	Yes	113	79
	No	30	21

*Abbreviation:* RCRSP = Rotator cuff-related shoulder pain.

**Table 3:** Chi-square tests.

Variable A	Variable B	Chi-Square	df	*p*-Value	Cramer’s V
Work setting	Surgery	0.008	1	0.928	
	Injection	0.000	1	0.997	
Level of post-graduate education	Surgery	2.031	1	0.154	
	Injection	0.120	1	0.729	
Age	Surgery	2.271	2	0.321	
	Injection	0.981	2	0.612	
Years working with RCRSP patients	Surgery	2.223	4	0.695	
	Injection	4.803	4	0.308	
Years qualified	Surgery	2.610	4	0.625	
	Injection	1.060	4	0.901	
Special interest in rotator cuff pathologies	Surgery	4.937	1	0.026	0.186
	Injection	9.143	1	0.002	0.253

*Abbreviation:* RCRSP = Rotator cuff-related shoulder pain.

**Table 4:** Parameter estimates for binary logistic regression.

Parameter	B	Sig.	Exp(B)	95% Wald Confidence Interval for Exp(B)	
				Lower	Upper
(Intercept)	−2.966	0.012	0.051	0.005	0.514
[Injection = 1 (Yes/Unsure)]	0.937	0.087	2.553	0.872	7.477
[Injection = 2 (No)]	0		1		
[Surgery = 1(Yes/Unsure)]	1.938	<0.001	6.944	2.447	19.706
[Surgery = 2 (No)]	0		1		
[Work setting = 1 (Other)]	1.619	0.014	5.049	1.386	18.394
[Work setting = 2 (Private)]	0		1		
[Level of education = 1 (PhD/MSc)]	−1.029	0.126	0.357	0.096	1.334
[Level of education = 2 (Seminar/None)]	0		1		
[Age = 1 [18–34]]	3.763	0.084	43.076	0.603	3078.942
[Age = 2 [35–44]]	1.399	0.435	4.051	0.121	135.916
[Age = 3 (>44)]	0		1		
[Years working with RCRSP patients = 1 [1–5]]	0.54	0.732	1.716	0.078	37.821
[Years working with RCRSP patients = 2 [6–10]]	2.364	0.144	10.634	0.445	254.251
[Years working with RCRSP patients = 3 [11–15]]	2.151	0.141	8.597	0.492	150.327
[Years working with RCRSP patients = 4 [16–20]]	1.037	0.513	2.822	0.126	63.235
[Years working with RCRSP patients = 5 (>20)]	0		1		
[Years qualified = 1 [1–5]]	−3.736	0.086	0.024	0	1.708
[Years qualified = 2 [6–10]]	−4.574	0.067	0.01	7.76 × 10^−5^	1.374
[Years qualified = 3 [11–15]]	−2.492	0.233	0.083	0.001	4.987
[Years qualified = 4 [16–20]]	−3.234	0.086	0.039	0.001	1.586
[Years qualified = 5 [21–25]]	0		1		
[Interest in shoulder pain = 1 (Yes)]	0.066	0.918	1.068	0.303	3.771
[Interest in shoulder pain = 2 (No)]	0		1		

*Abbreviation:* RCRSP = Rotator cuff-related shoulder pain.

**Table 5:** Proportion of recommended education topics.

	Frequency (n)	Percentage (%)
Pathology of RCRSP pain	111	78
Relationship between tendinopathy and pain	90	63
Risk factors	109	76
Factors influencing pain	93	65
Recommended physiotherapy management	103	72
Activity modification	116	81
Timeframe/indication for imaging	55	38
Timeframe/indication for injection	18	13
Timeframe/indication for surgery	23	16
Other	6	4

*Abbreviation:* RCRSP = Rotator cuff-related shoulder pain.

**Table 6:** Exercise load and resistance intensity guidelines for patients.

	Frequency (n)	Percentage (%)
Start with a low-load	42	29
Start with a load at 60–70% of 1RM	5	3
Load intensity based on symptoms	61	43
Load based on the level of fatigue	6	4
Only exert enough load to maintain the quality of the movement and avoid compensation	15	10
Load based on their training goal	14	10

*Abbreviation:* 1RM = 1 repetition maximum.

**Table 7:** Exercise prescription: guidelines for repetitions and sets.

	Frequency (n)	Percentage (%)
Specific sets and repetitions for everyone	9	6
Adapted to the patient’s symptoms and irritability	110	77
Based on the treatment goal	19	13
Other topics	5	3

**Table 8:** Exercise modification recommendations: load, sets, repetitions, and range of motion.

	Frequency (n)	Percentage (%)
Daily exercise	45	31
Performing the exercises 3–5 times per day	20	14
Several times per week (3–5 times per week)	20	14
Depending on the patient’s symptoms	43	30
Depending on the treatment goal (e.g., strengthening, hypertrophy, etc.)	13	9
Depending on fatigue levels	0	0
Other topics	2	1

**Table 9:** Recommendations for adjusting exercise load, sets, and range of motion.

	Frequency (n)	Percentage (%)
Suggest increasing/decreasing the load	71	50
Suggest increasing/decreasing the number of sets/repetitions	37	26
Suggest increasing/decreasing the range of motion	22	15
Other topics	13	9

## Data Availability

The data generated and analysed during the current study are available from the corresponding author upon reasonable request.

## Supplementary Materials

The following supporting information can be downloaded at https://www.mdpi.com/article/10.3390/clinpract16010011/s1. Supplementary File S1, Supplementary File S2, Supplementary File S3. References [16,18,19,24,25,26,27,28,46] are cited in the supplementary materials.

## Abbreviations

The following abbreviations are used in this manuscript:

RCRSP	Rotator Cuff-Related Shoulder Pain
MRI	Magnetic Resonance Imaging
US	Ultrasound
VAS	Visual Analogue Scale
