# Supplementary material for: Rotator Cuff-Related Shoulder Pain: A Survey of Current Physiotherapy Practice in Cyprus
Source: Clin Pract. 2026 Jan 4;16(1):11. doi: 10.3390/clinpract16010011 (PMC12839916; doi:10.3390/clinpract16010011)
Supplement: Supplementary file 1 [file clinpract-16-00011-s001.zip › Supplementary File S1.pdf]

**Supplementary File 1:** Survey instrument (Greek language).

# Έρευνα απευθύνεται σε Φυσικοθεραπευτές

Φυσικοθεραπευτική διαχείριση του πόνου στο μυϊκό στροφικό πέταλο της ωμικής ζώνης από Κύπριους Φυσικοθεραπευτές: Μελέτη Παρατήρησης (αριθμός έγκρισης ΕΕΒΚ ΕΠ 2024.01.172)

* Required

## Δήλωση συγκατάθεσης

Η παρακάτω έρευνα πραγματοποιείται υπό την αιγίδα του Τμήματος Φυσικοθεραπείας του Ευρωπαϊκού Πανεπιστημίου Κύπρου με υπεύθυνο ερευνητή τον Δρ. Γιώργος Παμπόρη και απευθύνεται σε Επαγγελματίες Φυσικοθεραπευτές μόνο.

Εισαγωγή: Ο πόνος που σχετίζεται με το στροφικό πέταλο του ώμου (Rotator Cuff Related Shoulder Pain) είναι ένας πολύ κοινός τύπος πόνου στον ώμο. Αυτή η κατάσταση είναι παρούσα στο 70% περίπου των ασθενών που εμφανίζουν πόνο στον ώμο. Ο πόνος που σχετίζεται με το στροφικό πέταλο του ώμου είναι ένας γενικός όρος που περιλαμβάνει ένα φάσμα παθήσεων του ώμου, συμπεριλαμβανομένου του υποακρωμιακού πόνου, του συνδρόμου (πρόσκρουσης), της τενοντοπάθειας του στροφικού πετάλου και των συμπτωματικών ρήξεων του στροφικού πετάλου. Ο πόνος που σχετίζεται με το μυϊκό στροφικό πέταλο προκαλεί μείωση της λειτουργικότητας και έως και το 50% των ατόμων που επηρεάζονται, θα εμφανίσουν πόνο πέραν των 12 μηνών. Υπάρχουν κάποιες ενδείξεις ότι τα άτομα με αυτή την πάθηση δε λαμβάνουν πάντα τις θεραπείες που απαιτούνται. Αυτή η έρευνα θα προσπαθήσει να ανακαλύψει εάν αυτό είναι αλήθεια και πόσο συχνά συμβαίνει στην Κυπριακή επικράτεια.

Σκοπός: Ο σκοπός αυτής της έρευνας είναι να αξιολογήσει τις θεραπείες που συστήνουν/πραγματοποιούν οι φυσικοθεραπευτές στην Κύπρο για πόνο σχετιζόμενο με το στροφικό πέταλο του ώμου αλλά και να ερευνήσει το κατά πόσο τις εφαρμόζουν. Ο στόχος αυτής της έρευνας είναι να διερευνήσει εάν οι φυσικοθεραπευτές τηρούν τη συνιστώμενη φροντίδα για τον πόνο που σχετίζεται με το μυϊκό στροφικό πέταλο.
Χρειάζονται περίπου 10 λεπτά για να ολοκληρωθεί το παρακάτω ερωτηματολόγιο και αποτελείται από ερωτήσεις πολλαπλής επιλογής και κλινικά σενάρια.

Συναίνεση συμμετοχής: Διαβάστε πλήρως αυτήν την Επεξηγηματική Δήλωση προτού αποφασίσετε εάν θα συμμετάσχετε ή όχι σε αυτήν την έρευνα. Εάν θέλετε περισσότερες πληροφορίες σχετικά με οποιαδήποτε πτυχή αυτού του έργου, σας ενθαρρύνουμε να επικοινωνήσετε με τους ερευνητές.

Τι περιλαμβάνει η έρευνα; Η έρευνα περιλαμβάνει τη συμπλήρωση αυτής της διαδικτυακής έρευνας. Όλες οι πληροφορίες από την έρευνα θα είναι εντελώς ανώνυμες, επομένως κανείς δε θα μπορεί να σας αναγνωρίσει.

Συναίνεση για συμμετοχή στο έργο και αποχώρηση από την έρευνα: Για να συμμετάσχετε στο ερευνητικό έργο το μόνο που χρειάζεται να κάνετε είναι να συμπληρώσετε τις ερωτήσεις. Απαντώντας στις ερωτήσεις και υποβάλλοντας το ερωτηματολόγιο συναινείτε στην έρευνα. Κάντε αυτό μόνο εάν είστε πρόθυμοι να λάβετε μέρος και συμφωνείται στα παρακάτω:
- Έχετε διαβάσει όλες τις παραπάνω πληροφορίες
- Έχετε συμφωνήσει ελεύθερα να συμμετάσχετε
- Είστε ενήλικες πτυχιούχοι Φυσικοθεραπευτές.

Πιθανά οφέλη και κίνδυνοι για τους συμμετέχοντες: Η συμμετοχή σας θα μας βοηθήσει να κατανοήσουμε τις θεραπείες που λαμβάνουν τα άτομα με πόνο που σχετίζεται με το στροφικό πέταλο και να κατευθύνουμε τελικά τις θεραπείες καλύτερα σε αυτήν την ομάδα ασθενών. Δεν υπάρχουν κίνδυνοι με τη συμμετοχή σας στην παρούσα έρευνα.

Αποθήκευση δεδομένων: Τα ηλεκτρονικά δεδομένα θα αποθηκευτούν σε έναν υπολογιστή με κωδικό πρόσβασης τα οποία θα αποθηκευτούν για 2 χρόνια. Μετά από αυτό το σημείο τα ηλεκτρονικά δεδομένα θα διαγραφούν οριστικά.

Αποτελέσματα: Τα αποτελέσματα του ερευνητικού έργου θα παρουσιαστούν μέσω άρθρου σε επιστημονικό περιοδικό και σε συνέδρια.

Παράπονα: Εάν έχετε οποιεσδήποτε ανησυχίες ή παράπονα σχετικά με τη διεξαγωγή του έργου, μπορείτε να το κάνετε μέσω της Διαδικασίας Παραπόνων του Πανεπιστημίου. Επικοινωνήστε με τον Καθηγητή Μάριο Βρυωνίδη , Αντιπρύτανη Έρευνας και Εξωτερικών Υποθέσεων, Email: m.vryonides@euc.ac.cy / Τηλέφωνο: 22713112

## Section

1. Παρακαλούμε δηλώστε την ηλικία σας: *

   - 18-24
   - 25-34
   - 35-44
   - 45-54
   - 55-64
   - 65-74

2. Παρακαλούμε δηλώστε το φύλο σας: *

   - Άνδρας
   - Γυναίκα
   - Προτιμώ να μην απαντήσω

3. Πόσο καιρό σε έτη ασκείτε το επάγγελμα του φυσικοθεραπευτή; *

   - 0-5
   - 6-10
   - 11-15
   - 16-20
   - 21-25
   - 26-30
   - 31 και πάνω

4. Πόσα χρόνια ασχολείστε με την κλινική φροντίδα ασθενών με πόνο στην περιοχή του ώμου; *

- 0-5
- 6-10
- 11-15
- 16-20
- 21-25
- 26-30
- 31 και πάνω

5. Σε ποια επαρχία εργάζεστε; *

- Λευκωσία
- Λεμεσός
- Λάρνακα
- Πάφος
- Αμμόχωστος

6. Πόσους ασθενείς με πρόβλημα στον ώμο παρακολουθείτε κατά μέσο όρο ανά μήνα; *

- <5
- 6-10
- 11-20
- 21-30
- >30

7. Επιλέξτε πως περιγράφεται καλύτερα το εργασιακό σας περιβάλλον: *

- ◯ Ιδιωτικό φυσικοθεραπευτήριο
- ◯ Δημόσια μονάδα υγείας (π.χ. κέντρο υγείας)
- ◯ Μονάδα φροντίδας ηλικιωμένων
- ◯ Νοσοκομείο
- ◯ Εκπαιδευτικό ίδρυμα (Σχολική μονάδα, Πανεπιστήμιο)
- ◯ Άλλο

8. Τι είδους κλινικά περιστατικά παρακολουθείτε; *

- ◯ Μυοσκελετικά
- ◯ Μυοσκελετικά και άλλα
- ◯ Δεν ασχολούμαι με μυοσκελετικά
- ◯ Δε δουλεύω σε κλινικό επίπεδο

9. Παρακαλούμε επιλέξτε οποιαδήποτε μεταπτυχιακή εκπαίδευση έχετε ολοκληρώσει, συμπληρώνοντας τον τομέα μελέτης δίπλα από την/τις απάντηση/εις: *

- ◯ Καμία
- ◯ Σεμινάριο-Πρόγραμμα Επιμόρφωσης
- ◯ Μεταπτυχιακό (MSc)
- ◯ Διδακτορικό (PhD)

10. Έχετε ιδιαίτερο ενδιαφέρον για περιστατικά πόνου στον ώμο ή πόνου σχετιζόμενου με το πέταλο των στροφέων; *

- ◯ Ναι
- ◯ Όχι

## Κλινικό περιστατικό

Άντρας 57 χρονών, λογιστής, παρουσιάζει 6μηνο ιστορικό ενόχλησης στην προσθιοπλάγια περιοχή του δεξιού ώμου. Ο πόνος αναπτύχθηκε σταδιακά, χωρίς ιστορικό τραύματος. Είναι διακοπτόμενος και επιδεινώνεται σε δραστηριότητες πάνω από το ύψος του ώμου, και κατά τον ύπνο στην πάσχουσα πλευρά. Ο ασθενής δεν αναφέρει πόνο σε κατάσταση ηρεμίας. Το παθητικό εύρος κίνησης της άρθρωσης του ώμου είναι φυσιολογικό. Η αξιολόγηση της αυχενικής μοίρας είναι φυσιολογική χωρίς συμπτώματα. Δεν έχουν πραγματοποιηθεί διαγνωστικές απεικονίσεις. Δεν υπάρχουν συνοδά προβλήματα, δε λαμβάνει κάποια φαρμακευτική αγωγή και δεν υπάρχουν ενδείξεις για "κόκκινες σημαίες" (red flags). Εκτός από τη σύσταση του προσωπικού του ιατρού να αποφεύγει δραστηριότητες που επιδεινώνουν τα συμπτώματα, δεν έχει λάβει καμία θεραπεία.

Για τον σκοπό αυτής της έρευνας, ορίζουμε τη συγκεκριμένη περίπτωση ως ως **πόνος στον ώμο που σχετίζεται με το στροφικό πέταλο**.

Ωστόσο, σημειώστε ότι στη βιβλιογραφία υπάρχουν πολλαπλοί συνώνυμοι όροι, όπως *τενοντοπάθεια του στροφικού πετάλου, τενοντοπάθεια υπερακανθίου, υπακανθίου ή υποπλατίου, τενοντίτιδα πετάλου των στροφέων, τραυματισμός ή ρήξη στο πέταλο των στροφέων, σύνδρομο του στροφικού πετάλου, σύνδρομο υπακρωμιακής πρόσκρουσης, σύνδρομο υπακρωμιακής στένωσης, σύνδρομο εξόδου υπακρωμιακής προστριβής ή σύνδρομο επώδυνου τόξου.*

11. Αναφερόμενοι στο παραπάνω περιστατικό, θα συνιστούσατε τη λήψη κάποιας διαγνωστικής απεικόνισης; *

◯ Ναι

◯ Όχι

12. Τι είδους διαγνωστική απεικόνιση θα συστήνατε; (Μπορείτε να επιλέξετε πολλαπλές απαντήσεις) *

☐ Ακτινογραφία

☐ Υπέρηχος

☐ Μαγνητική τομογραφία

☐ Η απεικόνιση δεν είναι αναγκαία

13. Εάν στις απαντήσεις της παραπάνω ερώτησης συμπεριλάβατε την επιλογή της ακτινογραφίας, αναφέρετε παρακάτω ποιες είναι οι κλινικές ενδείξεις για τη λήψη ακτινογραφίας; Εάν δεν συμπεριλάβατε την επιλογή ακτινογραφίας αναγράψτε ''Δεν απαιτείται'' *

14. Εάν στις απαντήσεις της ερώτησης 12 συμπεριλάβατε την επιλογή του υπερήχου, να αναφέρετε παρακάτω ποιες είναι οι κλινικές ενδείξεις για τη λήψη υπερήχου; Εάν δεν συμπεριλάβατε την επιλογή υπερήχου αναγράψτε ''Δεν απαιτείται'' *

15. Εάν στις απαντήσεις της ερώτησης 12 συμπεριλάβατε την επιλογή της μαγνητικής τομογραφίας, αναφέρετε παρακάτω ποιες είναι οι κλινικές ενδείξεις για τη λήψη μαγνητικής τομογραφίας; Εάν δεν συμπεριλάβατε την επιλογή της μαγνητικής τομογραφίας αναγράψτε ''Δεν απαιτείται'' *

16. Εάν στις απαντήσεις της ερώτησης 12 συμπεριλάβατε την επιλογή "Άλλο", να αναφέρετε την διαγνωστική απεικόνιση που θα προτείνατε και τις κλινικές ενδείξεις για την επιλογή σας. Εάν δε συμπεριλάβατε την επιλογή "Άλλο" αναγράψτε ''Δεν απαιτείται'' *

17. Τι είδους εκπαίδευση θα παρείχατε τυπικά σε έναν ασθενή με τη συγκεκριμένη παθολογία; (Μπορείτε να επιλέξετε πολλές απαντήσεις) *

- [ ] Πληροφορίες σχετικά με την παθολογία του πόνου του πετάλου των στροφέων, συμπεριλαμβανομένων των ιστών που πιθανά να σχετίζονται
- [ ] Τη σχέση ανάμεσα στην εμφάνιση τενοντοπάθειας του πετάλου των στροφέων και εμφάνισης πόνου
- [ ] Παράγοντες κινδύνου, όπως αλλαγή δραστηριοτήτων, ανύψωση μεγάλου βάρους, ηλικία, μεταβολισμός κ.ά.
- [ ] Παράγοντες που μπορεί να επηρεάσουν τον πόνο, όπως τα επίπεδα άγχους και οι πεποιθήσεις/προσδοκίες του ασθενούς
- [ ] Προτεινόμενη φυσικοθεραπευτική διαχείριση (διαχείριση παθολογίας με φυσικοθεραπευτικά μέσα)
- [ ] Τροποποίηση δραστηριοτήτων και στάσεων σώματος (πχ. εργασία, άθληση) εάν είναι επώδυνες
- [ ] Χρονικά στάδια εξέλιξης της παθολογίας και ενδείξεις για λήψη απεικόνισης
- [ ] Χρονικά στάδια εξέλιξης της παθολογίας και ενδείξεις για λήψη ενέσιμης θεραπείας
- [ ] Χρονικά στάδια εξέλιξης της παθολογίας και ενδείξεις για λήψη χειρουργικής αποκοτάστασης
- [ ] Άλλο

18. Τι πρόγραμμα ασκήσεων θα προτείνατε για τον συγκεκριμένο ασθενή; (Μπορείτε να επιλέξετε πολλές απαντήσεις) *

- [ ] Καθόλου άσκηση
- [ ] Διατάσεις
- [ ] Ισομετρικές ασκήσεις για τον ώμο
- [ ] Ισοτονικές ασκήσεις για τον ώμο
- [ ] Έκκεντρες ασκήσεις για τον ώμο
- [ ] Ισοκινητικές ασκήσεις για τον ώμο
- [ ] Ειδικές ασκήσεις για την ωμοπλάτη
- [ ] Ασκήσεις ιδιοδεκτικότητας
- [ ] Ειδικές ασκήσεις για τους μύες του πετάλου των στροφέων
- [ ] Ασκήσεις για την αυχενική και θωρακική μοίρα της σπονδυλικής στήλης
- [ ] Ασκήσεις για την κινητική αλυσίδα ολόκληρου του άνω άκρου
- [ ] Αερόβια άσκηση
- [ ] Άλλο

19. Ποιες άλλες στρατηγικές διαχείρισης θα συστήνατε για τον συγκεκριμένο ασθενή; (Μπορείτε να επιλέξετε πολλές απαντήσεις) *

- [ ] Συμβουλευτική για την λήψη παρακεταμόλης και αντιφλεγμονωδών φαρμάκων για τον πόνο
- [ ] Τεχνικές ανάταξης (manipulations)
- [ ] Κινητοποίηση αρθρώσεων (joint mobilization)
- [ ] Μάλαξη
- [ ] Θεραπεία προσανατολισμένη στην αυχενική/θωρακική μοίρα της σπονδυλικής στήλης
- [ ] Taping
- [ ] Βελονισμός/τεχνική ξηρής βελόνας
- [ ] Ηλεκτροθεραπεία (υπέρηχοι, TENS, παρεμβαλλόμενα ρεύματα κλπ)
- [ ] Θερμοθεραπεία ή κρυοθεραπεία
- [ ] Ανάπαυση-ξεκούραση
- [ ] Άλλο

20. Θα σκεφτόσασταν να παραπέμψετε τον ασθενή αυτό σε κλινικό ιατρό, ως κατάλληλο υποψήφιο για πιθανή ενέσιμη θεραπεία; *

- Ναι
- Όχι
- Δεν είμαι σίγουρος/η

21. Εάν στην ερώτηση 20 απαντήσατε "Ναι" αναφέρετε παρακάτω ποιες είναι οι κλινικές ενδείξεις για ενέσιμη θεραπεία. Εάν απαντήσατε "Όχι" ή "Δεν είμαι σίγουρος/η" αναγράψτε "Δεν απαιτείται" *

22. Θα σκεφτόσασταν να παραπέμψετε αυτόν τον ασθενή σε ορθοπαιδικό χειρουργό, ως κατάλληλο υποψήφιο για πιθανή χειρουργική αντιμετώπιση; *

- Ναι
- Όχι
- Δεν είμαι σίγουρος/η

23. Εάν στην ερώτηση 22 απαντήσατε "Ναι" να αναφέρετε παρακάτω ποιες είναι οι κλινικές ενδείξεις για χειρουργική αντιμετώπιση. Εάν απαντήσατε "Όχι" ή "Δεν είμαι σίγουρος/η" αναγράψτε "Δεν απαιτείται" *

24. Κατά τη διαμόρφωση προγραμμάτων άσκησης, τι οδηγίες δίνετε, συνήθως, όσον αφορά τον πόνο κατά τη διάρκεια της άσκησης; *

- Να μην πονάει καθόλου όταν εκτελεί ασκήσεις
- Ο πόνος επιτρέπεται κατά την εκτέλεση ασκήσεων
- Ο πόνος να μην ξεπερνάει το 2-3 στα 10 στην κλίμακα VAS (0-10)
- Ο πόνος να μην ξεπερνάει το 6-7 στα 10 στην κλίμακα VAS (0-10)
- Ο πόνος να υποχωρεί μετά το τέλος της άσκησης
- Ο πόνος να υποχωρεί την επόμενη ημέρα (εντός 24 ωρών)

25. Κατά τη συνταγογράφηση ασκησιολογίου, τι οδηγίες δίνετε, συνήθως, όσον αφορά το φορτίο/επίπεδο αντίστασης; *

- ◯ Να ξεκινούν με ένα ήπιο φορτίο (π.χ. αλτήρα) 1-2 κιλών
- ◯ Να ξεκινούν με ένα φορτίο 60-70% της 1 μέγιστης επανάληψης (Repetition Maximum)
- ◯ Να καθορίζουν το φορτίο βάση συμπτωμάτων (π.χ. όποιο φορτίο οδηγεί σε πόνο όχι μεγαλύτερο του 4-5 στα 10 στην κλίμακα VAS)
- ◯ Να καθορίζουν το φορτίο βάση της κόπωσης που τους προκαλεί (π.χ. φορτίο που προκαλεί σημαντική κόπωση στις 12 επαναλήψεις-μέχρι αποτυχίας)
- ◯ Να καθορίζουν το φορτίο βάση του μέγιστου φορτίου που μπορούν να σηκώσουν χωρίς να επηρεάζεται αρνητικά η τεχνική τους κατά την εκτέλεση της άσκησης.
- ◯ Να καθορίζουν το φορτίο βάση του στόχου που έχουν (π.χ. άσκηση για δύναμη, υπερτροφία, αντοχή)

26. Κατά τη συνταγογράφηση ασκησιολογίου, τι οδηγίες δίνετε, συνήθως, όσον αφορά τις επαναλήψεις/σετ; *

- ◯ Συγκεκριμένο σετ και επαναλήψεις για όλους (π.χ. 3 σετ των 12 επαναλήψεων)
- ◯ Αναλόγως συμπτωμάτων και ευερεθιστότητας του ασθενή
- ◯ Αναλόγως του στόχου (π.χ. 3 × 45 sec κράτημα για ισομετρικές, 3 σετ × 12 επαναλήψεις για ισοτονικές)”
- ◯ Άλλο

27. Κατά τη συνταγογράφηση ασκησιολογίου, τι οδηγίες δίνετε, συνήθως, όσον αφορά τη συχνότητα; *

- ◯ Προτείνω καθημερινή εκτέλεση των ασκήσεων
- ◯ Προτείνω καθημερινή εκτέλεση των ασκήσεων 3-5 φορές την ημέρα
- ◯ Πολλές φορές μέσα στην εβδομάδα (3-5 φορές)
- ◯ Αναλόγως συμπτωμάτων, πόνου κτλ
- ◯ Αναλόγως στόχου (π.χ. ενδυνάμωση, υπερτροφία κτλ)
- ◯ Αναλόγως κόπωσης
- ◯ Άλλο

28. Κατά τη συνταγογράφηση ασκησιολογίου, τι οδηγίες δίνετε, συνήθως, όσον αφορά την πρόοδο ή τον περιορισμό της προόδου των ασκήσεων; *

- ◯ Προτείνω να αυξήσουν/μειώσουν το φορτίο
- ◯ Προτείνω να αυξήσουν/μειώσουν τα σετ και τις επαναλήψεις
- ◯ Προτείνω να αυξήσουν/μειώσουν το εύρος που εκτελούν την άσκηση
- ◯ Άλλο

29. Πώς παρέχετε, συνήθως, στους ασθενείς σας οδηγίες για την εκτέλεση των ασκήσεων "για το σπίτι" (άσκηση ή γενικές πληροφορίες); (Μπορείτε να επιλέξετε παραπάνω από μία απαντήσεις) *

- ☐ Γραπτές ή εκτυπωμένες πληροφορίες
- ☐ Συνδέσμους για διαδικτυακά βίντεο ή ιστοσελίδες
- ☐ Μαγνητοσκοπημένα βίντεο στο κινητό τους τηλέφωνο ή σε κάποια άλλη συσκευή
- ☐ Προφορικές οδηγίες

30. Με τι συχνότητα θα ελέγχατε και αντίστοιχα πιθανώς θα τροποποιούσατε το ασκησιολόγιο ενός ασθενούς με αναφερόμενο πόνο στο πέταλο των στροφέων; *

- ◯ Δεν θα συνταγογραφούσα ασκησιολόγιο
- ◯ Ποτέ ύστερα από την αρχική μου συνταγογράφηση
- ◯ Τουλάχιστον σε εβδομαδιαία βάση
- ◯ Περίπου κάθε 2 εβδομάδες
- ◯ Περίπου κάθε 3 εβδομάδες
- ◯ Περίπου μία φορά το μήνα ή και περισσότερο

31. Για πόσο διάστημα θα αναμένατε να χρειαστεί φυσικοθεραπείες ένας ασθενής με αναφερόμενο πόνο στο πέταλο των στροφέων; *

- ◯ Έως 3 εβδομάδες
- ◯ Έως 6 εβδομάδες
- ◯ Έως 8 εβδομάδες
- ◯ Έως 3 μήνες
- ◯ Έως 6 μήνες
- ◯ Έως 12 μήνες

32. Παρακαλούμε επιλέξτε τις 3 καλύτερες επιλογές βάσει της προτίμησης σας όσον αφορά την επαγγελματική σας εξέλιξη για την παρακολούθηση κλινικών περιστατικών-ασθενών με πόνο σχετιζόμενο με το πέταλο των στροφέων. *

Please select 3 options.

- ☐ Συμμετοχή σε ένα επαγγελματικό συνέδριο/διάλεξη ως εκπρόσωπος (κυρίως διάλεξη)
- ☐ Δια ζώσης συμμετοχή σε εργαστηριακό σεμινάριο
- ☐ Προσωπική μελέτη (πχ βιβλίων, άρθρων)
- ☐ Διαδραστική διαδικτυακή μάθηση
- ☐ Συνδυασμός διαδικτυακού και διαπροσωπικού τρόπου μάθησης
- ☐ Επαγγελματικού χαρακτήρα φόρουμ στα μέσα κοινωνικής δικτύωσης
