# Supplementary material for: Rotator Cuff-Related Shoulder Pain: A Survey of Current Physiotherapy Practice in Cyprus
Source: Clin Pract. 2026 Jan 4;16(1):11. doi: 10.3390/clinpract16010011 (PMC12839916; doi:10.3390/clinpract16010011)
Supplement: Supplementary file 1 [file clinpract-16-00011-s001.zip › Supplementary File S2.pdf]

Supplementary file 2: Survey instrument (English translation).

Please note: this English language version is a direct translation of the original Greek questionnaire.

Rotator cuff-related shoulder pain: A survey of current (2024) physiotherapy practice in Cyprus

Survey for Physiotherapists

Physiotherapeutic Management of Rotator Cuff-Related Shoulder Pain by Cypriot Physiotherapists: An Observational Study (Approval Number: ΕΕΒΚ ΕΠ 2024.01.172)

* Required

Declaration of consent

The following research is conducted under the auspices of the Department of Physiotherapy at the European University Cyprus, led by Dr. George M. Pamboris, and is intended exclusively for Registered Physiotherapists.

Introduction: Rotator Cuff-Related Shoulder Pain (RCRSP) is one of the most common causes of shoulder pain, affecting approximately 70% of patients experiencing shoulder discomfort. This term is an umbrella diagnosis encompassing various shoulder conditions, including subacromial pain syndrome (impingement), rotator cuff tendinopathy, and symptomatic rotator cuff tears. Pain associated with rotator cuff dysfunction often leads to reduced function, with up to 50% of affected individuals experiencing symptoms beyond 12 months. Evidence suggests that patients with this condition do not always receive appropriate treatment. This research aims to investigate whether this is the case in Cyprus and assess the frequency and extent of inadequate treatment in this population.

Purpose: The purpose of this research is to evaluate the treatments recommended and performed by physiotherapists in Cyprus for rotator cuff-related shoulder pain and to assess the extent to which these treatments are applied in clinical practice. This study aims to determine whether physiotherapists adhere to recommended care guidelines for managing rotator cuff pain. The questionnaire below takes approximately 10 minutes to complete and consists of multiple-choice questions and clinical scenarios.

Consent to Participate: Please read this Explanatory Statement carefully before deciding whether to participate in this research. If you require further information about any aspect of this study, please feel free to contact the researchers.

What does the survey involve? This research involves completing an online survey. All responses will remain completely anonymous, ensuring that no participant can be identified.

Consent to Participate and Withdraw: To participate in this research project, simply complete and submit the questionnaire. By doing so, you are providing your informed consent to take part in the survey. Please proceed only if you are willing to take part and agree to the following:
- Have read and understood all of the above information;
- Freely agreed to participate;
- Are a qualified adult physiotherapist.

Potential Benefits and Risks to Participants: Your participation will contribute to a better understanding of the treatments provided for individuals with rotator cuff-related pain, ultimately helping to improve targeted treatment strategies for this patient group. There are no known risks associated with participating in this research.

Data Storage: All electronic data will be securely stored on a password-protected computer for a period of two years. After this time, the data will be permanently deleted.

Results: The findings of this research project will be published in a scientific journal and presented at conferences.

The results of the research project will be presented through an article in a scientific journal and at conferences.

If you have any concerns or complaints regarding the conduct of this research project, you may submit them through the University's Complaints Procedure. For assistance, please contact Professor Marios Vryonides, Vice-Rector for Research and External Affairs, via email at m.vryonides@euc.ac.cy / phone at 22713112.

1. Please state your current age: *

- ◯ 18-24
- ◯ 25-34
- ◯ 35-44
- ◯ 45-54
- ◯ 55-64
- ◯ 65-74

2. Please state your gender: *

- ◯ Man
- ◯ Woman
- ◯ I prefer not to answer

3. How long have you been working as a physical therapist (in years)? *

- ◯ 0-5
- ◯ 6-10
- ◯ 11-15
- ◯ 16-20
- ◯ 21-25
- ◯ 26-30
- ◯ >31

4. How many years have you been involved in the clinical care of patients with pain in the shoulder area? *

- ◯ 0-5
- ◯ 6-10
- ◯ 11-15
- ◯ 16-20
- ◯ 21-25
- ◯ 26-30
- ◯ >31

5. In which district do you work? (If you work outside of Cyprus, do not continue filling out the questionnaire). *

- ◯ Nicosia
- ◯ Limassol
- ◯ Larnaca
- ◯ Paphos
- ◯ Famagusta

6. How many patients with shoulder complaints do you treat in an average month? *

- ◯ < 5
- ◯ 6-10
- ◯ 11-20
- ◯ 21-30
- ◯ >30

7. Choose how your work environment is best described: *

- ☐ Private physical therapy clinic
- ☐ Public health facility (e.g., health center)
- ☐ Elderly care unit
- ☐ Hospital
- ☐ Educational institution
- ☐ Other

8. Which patient clientele do you work with? *

- ○ Patients with musculoskeletal diseases
- ○ Patients with musculoskeletal diseases and patients with other diseases
- ○ Patients with non-musculoskeletal diseases
- ○ I am not currently working clinically

9. Please select any postgraduate education/training you have completed by filling in the field of study next to the answer(s): *

- ◯ None
- ◯ Seminar-Training
- ◯ Master (MSc)
- ◯ Doctorate (PhD)

10. Do you have a particular interest in shoulder pain or rotator cuff-related pain? *

- ◯ Yes
- ◯ No

Clinical case

A 57-year-old man, an accountant, presents with a 6-month history of discomfort in his right anterolateral shoulder region. The pain developed gradually and there is no history of trauma. The pain is intermittent, aggravated by reaching overhead and sleeping on his affected side. The patient has no pain at rest. Passive range of motion of the shoulder joint is normal. Cervical spine assessment is normal. No imaging studies have been performed. The patient has no other known medical conditions, is not taking any medication, and shows no indications of "red flags". Aside from advice from his GP to avoid aggravating activities, the patient has not had any treatment.

For the purposes of this survey, the health problem described is defined as **rotator cuff-related shoulder pain**.

However, please note that there are several synonyms in the literature, such as rotator cuff tendinopathy, supraspinatus, infraspinatus or subscapularis tendinopathy, rotator cuff tendinitis, rotator cuff injury, rotator cuff tear, rotator cuff syndrome, subacromial impingement syndrome, subacromial tightness syndrome, supraspinatus outlet syndrome, or painful arc syndrome.

11. Would you recommend an imaging examination in relation to this case? *

- ◯ Yes
- ◯ No

12. What kind of diagnostic imaging would you recommend? (You can choose multiple answers) *

- ☐ X-ray
- ☐ Ultrasound
- ☐ Magnetic resonance
- ☐ Illustration is not necessary

13. If you included the option of radiography in your answers to the above question, please indicate below what the clinical indications for radiography are. If you did not include the x-ray option, write "Not Required" *

14. If you included the option of ultrasound in your answers to question 12, please indicate below what the clinical indications for ultrasound are. If you did not include the ultrasound option, write "Not required" *

15. If you included the option of MRI in your answers to question 12, please indicate below what the clinical indications for obtaining an MRI are. If you did not include the MRI option, write "Not Required" *

16. If you answered "Other" in your answers to question 12, please indicate the diagnostic imaging you would recommend and the clinical indications for your choice. If you did not include the "Other" option, write "Not Required" *

17. What type of educational topics would you typically provide with this patient group? (You can choose multiple answers) *

- [ ] Pathology of rotator cuff-related shoulder pain, including the tissues that may be involved
- [ ] Relationship between tendinopathy and pain
- [ ] Risk factors, such as changes in activities, lifting heavy weights, age, metabolic disorders, etc.
- [ ] Factors that may influence pain, such as stress and beliefs/expectations
- [ ] Recommended physical therapy management (pathology management with physical therapy modalities)
- [ ] Adjustment of activities and postures (at work, during sports) if painful
- [ ] Timing and indication for imaging
- [ ] Timing and indication for injection
- [ ] Timing and indication for surgery
- [ ] Other

18. What exercise strategies would you usually recommend for this patient? (You can choose multiple answers) *

- [ ] No exercise
- [ ] Stretching
- [ ] Isometric shoulder exercises
- [ ] Isotonic shoulder exercises
- [ ] Eccentric shoulder exercises
- [ ] Isokinetic exercises for the shoulder
- [ ] Specific exercises for the scapula
- [ ] Proprioceptive exercises
- [ ] Specific exercise for the rotator cuff musculature
- [ ] Cervical and/or thoracic spine exercises
- [ ] Global exercise for upper limb kinetic chain
- [ ] Aerobic exercise
- [ ] Other

19. What other management strategies would you recommend for this patient? (You can choose multiple answers) *

- [ ] Advice to take paracetamol and/or NSAIDs
- [ ] Manipulation
- [ ] Mobilization
- [ ] Massage
- [ ] Treatment directed to the cervical/thoracic spine
- [ ] Taping
- [ ] Acupuncture/dry needling
- [ ] Electrotherapy (ultrasound, TENS, interferential current, etc.)
- [ ] Heat or cold therapy
- [ ] Rest
- [ ] Other

20. Would you consider referring this patient to a clinician as a suitable candidate for possible injectable treatment? *

- ○ Yes
- ○ No
- ○ I am not sure

21. If you answered "Yes" to question 20, state below what the clinical indications for injection therapy are. If you answered "No" or "Not sure", write "Not required" *

22. Would you consider referring this patient to an orthopedic surgeon as a suitable candidate for possible surgical management? *

- Yes
- No
- I am not sure

23. If you answered "Yes" to question 22, state below what the clinical indications for surgical treatment are. If you answered "No" or "Not sure", write "Not required" *

24. When you recommend exercises, what instructions do you usually give regarding pain during exercise? *

- ◯ No pain at all during exercise
- ◯ Some pain is acceptable during exercise
- ◯ Pain should not exceed 2-3/10 on the VAS scale (0-10)
- ◯ Pain should not exceed 6-7/10 on the VAS scale (0-10) Pain
- ◯ should subside after the exercise session
- ◯ Pain should subside by the next day (within 24 hours)

25. When prescribing an exercise program, what guidelines do you usually give in terms of load/resistance level? *

- ◯ Start with a low load (e.g., dumbbell) of 1-2 kg
- ◯ Start with a load at 60-70% of 1 RM
- ◯ Load based on symptoms (e.g., any load that results in pain no greater than 4-5/10 on the VAS scale)
- ◯ Load based on the level of fatigue (e.g., load that causes significant fatigue at 12 repetitions to failure)
- ◯ Only exert enough load to maintain the quality of the movement and avoid compensation
- ◯ Load based on their training goal (e.g., exercise for strength, hypertrophy, endurance).

26. When prescribing an exercise program, what instructions do you usually give in terms of reps/sets? *

- ◯ Specific set and repetitions for everyone
- ◯ Adapted to patient's symptoms and irritability
- ◯ Based on the treatment goal
- ◯ Other

27. When prescribing an exercise regimen, what instructions do you usually give in terms of frequency? *

- ◯ Daily exercise
- ◯ Performing the exercises 3-5 times per day
- ◯ Several times per week (3-5 times per week)
- ◯ Depends on patient’s symptoms
- ◯ Depends on the treatment goal (e.g., strengthening, hypertrophy, etc.)
- ◯ Depends on fatigue
- ◯ Other

28. When prescribing an exercise program, what instructions do you usually give in terms of progressing or regressing the exercises? *

- ◯ Suggest increasing/decreasing the load
- ◯ Suggest increasing/decreasing the number of sets/repetitions
- ◯ Suggest increasing/decreasing the range of motion
- ◯ Other

29. How do you usually provide your patients with instructions on how to do the "home" exercises (exercise or general information)? (You can choose more than one answer) *

- ☐ Written or printed information
- ☐ Links to online videos or websites
- ☐ Recorded videos on the patient’s cell phone or other devices
- ☐ Verbal information

30. How often would you review and possibly adjust the exercise program of a patient with reported rotator cuff pain? *

- ◯ I would not prescribe any exercises
- ◯ Not at all since my original prescription
- ◯ At least weekly
- ◯ About every 2 weeks
- ◯ About every 3 weeks
- ◯ About once a month or more

31. How long would you expect a patient with reported rotator cuff pain to need physical therapy? *

- ◯ Up to 3 weeks
- ◯ Up to 6 weeks
- ◯ Up to 8 weeks
- ◯ Up to 3 months
- ◯ Up to 6 months
- ◯ Up to 12 months

32. Please select the top 3 options based on your preference for your career development in clinical case follow-up of patients with rotator cuff pain. *

Choose a maximum of 3 options.

- [ ] Attending a professional conference/lecture as a delegate (mainly lecture)
- [ ] Live participation in a laboratory seminar
- [ ] Personal study (e.g., books, articles)
- [ ] Interactive online learning
- [ ] Combination of online and face-to-face learning
- [ ] Professional social media forum
