# Supplementary material for: Rotator Cuff-Related Shoulder Pain: A Survey of Current Physiotherapy Practice in Cyprus
Source: Clin Pract. 2026 Jan 4;16(1):11. doi: 10.3390/clinpract16010011 (PMC12839916; doi:10.3390/clinpract16010011)
Supplement: Supplementary file 1 [file clinpract-16-00011-s001.zip › Supplementary File S3.pdf]

Supplementary File 3: Synthesis of best available evidence for clinical vignette (2024).

Clinical consideration	Recommended management for vignette	Rationale
Imaging	No imaging indicated	Imaging is not initially indicated in adult patients with limited movement and non-traumatic shoulder pain in the absence of red flags [1–3]. Imaging is only indicated in non-traumatic shoulder pain in the presence of red flag presentations, non-mechanical pain, and/or no response to an active treatment program after 4-6 weeks [1–4]. An MRI scan is indicated if no improvement has been achieved with conservative treatment or in the case of long-lasting unexplained pain, persistent weakness in shoulder elevation and rotation, or suspected rotator cuff rupture [1,5].
Referral for surgical opinion	No referral indicated	For non-traumatic rotator cuff disorders, surgical referral is indicated if functional deficits and activity impairments in everyday life or at work persist after 3 to 6 months of active participation in a rehabilitation program or after 3 to 12 weeks if functional impairment worsens or remains the same [1,2].
Referral for injection	No injection indicated	Heterogeneity in the timing and specific indication for corticosteroid injection for rotator cuff tendinopathy is evident in the guidelines. It is common sense is recommend injections only with severe pain and persisting pain beyond the conservative treatment period [2,4]. Because of the possible damaging effect on tendon tissue, a maximum of two corticosteroid injections are recommended [2,3].
Exercise therapy	An exercise program of at least a 12-week duration is indicated	Prescribed exercise programs are recommended as the initial treatment for rotator cuff tendinopathy [2–4,6,7]. Exercise programs should be undertaken for a minimum of 12 weeks but may need to be continued for 6-12 months [2–4,6].
Education	Education is indicated	Education is recommended as an integral component of rotator cuff tendinopathy management [2–4]. General information about treatment management in terms of shared decision making and the possible modification of stressful activities can be

		found in the overarching content [2]. Furthermore, health-related beliefs that can affect the patient's treatment outcomes, as well as information about relevant risk factors and indications of serious pathologies (red flags), can be identified as relevant to the conversation [2–4].
Adjunctive treatment	Manual therapy (mobilization and manipulation) can be used in conjunction with an active treatment approach	Manual therapy, when combined with an active program, shows short-term improvement in pain reduction in patients [1,2,4,8,9].
	Physical modalities such as US, TENS, low-level laser therapy, and bipolar interferential current are not indicated	Physical modalities including therapeutic ultrasound, transcutaneous electromagnetic stimulation, low-level laser therapy, and bipolar interferential current show no benefit for rotator cuff tendinopathy and are not indicated [2,8,9].
	Taping, acupuncture, myofascial trigger point therapy, and massage unclear evidence	The systematic review and meta-analysis by Steuri et al. [9] evaluated the evidence for the effectiveness of transcutaneous electrical nerve stimulation (TENS), acupuncture, myofascial trigger point therapy, and massage, concluding that it was insufficient to support clear clinical recommendations. Similarly, Haik et al. [8], in their systematic review, found no conclusive evidence regarding the efficacy of acupuncture, dry needling, or TENS. Additionally, the effectiveness of taping compared to sham taping in pain reduction was classified as exhibiting "uncertain treatment effects," largely due to poor study quality [8,9].

References

1. Doiron-Cadrin, P.; Lafrance, S.; Saulnier, M.; Cournoyer, É.; Roy, J.-S.; Dyer, J.-O.; Frémont, P.; Dionne, C.; MacDermid, J.C.; Tousignant, M.; et al. Shoulder rotator cuff disorders: A systematic review of clinical practice guidelines and semantic analyses of recommendations. *Arch. Phys. Med. Rehabil.* **2020**, *101*, 1233–1242. https://doi.org/10.1016/j.apmr.2019.12.017
2. Hopman, K.; Krahe, L.; Lukersmith, S.; McColl, A.; Vine, K. Clinical practice guidelines for the management of rotator cuff syndrome in the workplace: Technical report. In *Medicine, Rural Clinical School*; Krahe, L., Scahill, L., Eds.; University of New South Wales, Medicine, Rural Clinical School: Port Macquarie, Australia, 2013. Available online: https://rcs.med.unsw.edu.au/rotatorcuffsyndromeguidelines (accessed on 12 July 2024).
3. Kulkarni, R.; Gibson, J.; Brownson, P.; Thomas, M.; Rangan, A.; Carr, A.J.; Rees, J.L. Subacromial shoulder pain. *Shoulder Elb.* **2015**, *7*, 135–143. https://doi.org/10.1177/1758573215576456

4. Diercks, R.; Bron, C.; Dorrestijn, O.; Meskers, C.; Naber, R.; de Ruiter, T.; Willems, J.; Winters, J.; van der Woude, H.J. Guideline for diagnosis and treatment of subacromial pain syndrome. *Acta Orthop.* **2014**, *85*, 314–322. https://doi.org/10.3109/17453674.2014.920991
5. Weber, S.; Chahal, J. Management of Rotator Cuff Injuries. *J. Am. Acad. Orthop. Surg.* **2020**, *28*, E193–E201. https://doi.org/10.5435/JAAOS-D-19-00463
6. Codsi, M.; Howe, C.R. Shoulder conditions: Diagnosis and treatment guideline. *Phys. Med. Rehabil. Clin. N. Am.* **2015**, *26*, 467–489. https://doi.org/10.1016/j.pmr.2015.04.007
7. Pedowitz, R.A.; Yamaguchi, K.; Ahmad, C.S.; Burks, R.T.; Flatow, E.L.; Green, A.; Iannotti, J.P.; Miller, B.S.; Tashjian, R.Z.; Watters, W.C.; et al. Optimizing the management of rotator cuff problems. *J. Am. Acad. Orthop. Surg.* **2011**, *19*, 368–379. https://doi.org/10.5435/00124635-201106000-00007
8. Haik, M.N.; Alburquerque-Sendín, F.; Moreira, R.F.C.; Pires, E.D.; Camargo, P.R. Effectiveness of physical therapy treatment of clearly defined subacromial pain: A systematic review of randomised controlled trials. *Br. J. Sports Med.* **2016**, *50*, 1124–1134. https://doi.org/10.1136/bjsports-2015-095771
9. Steuri, R.; Sattelmayer, M.; Elsig, S.; Kolly, C.; Tal, A.; Taeymans, J.; Hilfiker, R. Effectiveness of conservative interventions including exercise, manual therapy and medical management in adults with shoulder impingement: A systematic review and meta-analysis of RCTs. *Br. J. Sports Med.* **2017**, *51*, 1340 LP–1347. https://doi.org/10.1136/bjsports-2016-096515
